# Fixed point results for ℑ-Contractions in JS-generalized metric spaces with an application

**DOI:** 10.1371/journal.pone.0314493

**Published:** 2025-02-18

**Authors:** Bilal Iqbal, Naeem Saleem, Maggie Aphane, Asima Razzaque

**Affiliations:** 1 Department of Mathematics, University of Management and Technology, Lahore, Pakistan; 2 Department of Mathematics and Applied Mathematics, Sefako Makgatho Health Sciences University, Ga-Rankuwa, Pretoria, South Africa; 3 Department of Basic Sciences, Preparatory Year, King Faisal University, Al-Ahsa, Saudi Arabia; 4 Department of Mathematics, College of Science, King Faisal University, Al-Ahsa, Saudi Arabia; University of Education, PAKISTAN

## Abstract

The goal of this work is to establish ℑ-contractions and to show some novel fixed point theorems for these contractive conditions in the setting of generalized metric spaces in the sense of Jleli and Samet. Finally, using proven fixed point results, an existence result for a solution of the RLC circuit’s current differential equation is established.

## 1 Introduction

The Banach fixed point theorem [1] served as the inspiration for metric fixed point theory. Because this approach has many applications in several disciplines, many authors have expanded it in many ways [2–5]. Wardowski [6] provides one such astonishing and significant generalization. He introduced the notion of ℑ-contraction as follows:

**Definition 0.1**. *Let* (*z*, *m*) *be a metric space. A mapping* ξ: *z* → *z* *is said to be an* ℑ-*contraction, if there is* ℑ ∈ Δ(ℑ) *and* λ > 0 *such that for all* x, y ∈ *z*

$$\begin{array}{c} \lambda +ℑ(m(\xi x,\xi y))\leq ℑ(m(x,y)), \end{array}$$ (1)

*where* Δ(ℑ) *is the family of all mappings* ℑ:(0,+∞)→R *obeying the following conditions*:

(ℑ_1_) ℑ(x) < ℑ(y) *for all* x < y;
(ℑ_2_) *for all sequences* {η_p_} ⊆ (0, + ∞), lim_p→∞_ η_p_ = 0, *if and only if* lim_p→∞_ ℑ(η_p_) = −∞;
(ℑ_3_) *there exists* 0 < ℓ < 1 *such that* ${\text{lim}}_{\eta \to 0^{+}}$ η^ℓ^ℑ(η) = 0.

Wardowski’s result is given as follows:

**THEOREM 0.1** [6]. *Let* (*z*, *m*) *be a complete metric space and* ξ: *z* → *z* *be* ℑ-*contraction. Then* x* ∈ *z is a unique fixed point of* ξ *and for each* x_0_ ∈ *z the sequence* ${\left\{\xi^{p}x_{0}\right\}}_{p\in N}$ *is convergent to* x*.

In [7], Secelean demonstrated that condition (ℑ_2_) may be overtaken by an equivalent and simpler one.

(${ℑ}_{2}^{\prime }$) ${\text{lim}}_{\text{t}\to 0^{+}}$ ℑ(t) = −∞.

**Lemma 0.1**. *Let* ℑ:(0,∞)→R *be a function obeying* (ℑ_1_) *and* (${ℑ}_{2}^{\prime }$), *then for all sequence* {t_p_} ⊆ (0, ∞)

$$\begin{array}{c} ℑ\left({\text{t}}_{p}\right)\to -\infty \;\text{implies}\;{\text{t}}_{p}\to 0. \end{array}$$

Following that, Piri and Kumam [8] established Wardowski’s theorem utilising (${ℑ}_{2}^{\prime }$) and the continuity of ℑ rather than (ℑ_2_) and (ℑ_3_), respectively. Wardowski [9] later proved a fixed point theorem for ℑ-contractions when λ is treated as a function. Recently, other authors demonstrated (in various methods) Wardowski’s original results in the absence of both criterias (ℑ_2_) and (ℑ_3_) (see, [10, 11]. To more in this direction, consult [12–15]. Very recently, Derouiche and Ramoul [16] used a relaxed version of (ℑ_2_) and also dropped (ℑ_3_) to establish some new fixed point results in the context of b-metric spaces.

On the other side, the concept of standard metric space is generalized in numerous ways (see [17–21]. Jleli and Samet [22] recapitulated a huge class of topological spaces by introducing the most prevailing generalizations of metric spaces namely JS-generalized metric spaces. More far, in [23], Karapinar *et al*. achieved fixed point theorems under very general contractive conditions and Altun *et al*. [24] proved a Feng-Liu’s type fixed point theorem in JS-generalized metric spaces. While, in [25], Dumitrescu and Pitea presented extensions of some classic results regarding the existence and uniqueness of fixed points of operators fulfilling generalized contractive conditions in the setting of JS-generalized metric spaces. Quite recently, Saleem *et al*. [26] proved some new fixed point theorems, coincidence point theorems and common fixed point theorems for multivalued ℑ-contractions in the framework of JS-generalized metric spaces. Afterwards, Iqbal *et al*. [27] derived the coincidence point and common fixed point results for ℑ-type mappings with regard to JS-generalized metric spaces.

A binary relation on *z* is a non-empty subset ${∼}_{R}$ of the Cartesian product *z* × *z*. For ease of use, we designate $\text{x}{∼}_{R}\text{y}$ if $(\text{x},\text{y})\in {∼}_{R}$. The concepts of antisymmetry, preorder, reflexivity, transitivity, and partial order can be found in [28]. The trivial preorder on *z* is denoted by ${∼}_{R_{z}}$, and is given by $\text{x}{∼}_{R_{z}}\text{y}$ for each x, y ∈ *z*. Here after, R and N demonstrate the set of real numbers and the set of non-negative integers, respectively. Let *z* be a non-empty set and J_d_: *z* × *z* → [0, ∞] be a given mapping. Following Jleli and Samet [22], for every x ∈ *z*, define the set

$$\begin{array}{c} c(J_{d},z,x)=\{\left\{x_{p}\right\}\subset z:\underset{p\to \infty }{\text{lim}}\;J_{d}\left(x_{p},x\right)=0\}. \end{array}$$ (2)

**Definition 0.2** [22]. *Let z be a non-empty set and* J_d_: *z* × *z* → [0, ∞] *be a function that complies with the following criteria for all* x, y ∈ *z*:

(J_d1_) J_d_(x, y) = 0 *implies* x = y;
(J_d2_) J_d_(x, y) = J_d_(y, x);
(J_d3_) *there exist* κ > 0 *such that* (x, y) ∈ *z* × *z*, {x_p_} ∈ J_d_, *z*, x) *implies*
  $$\begin{array}{c} J_{d}\left(x,y\right)\leq \kappa \underset{p\to \infty }{\text{lim}}\;\text{sup}\;J_{d}\left(x_{p},y\right). \end{array}$$ (3)

*Then* J_d_ *is called a JS-generalized metric and the pair* (*z*, J_d_) *is called a JS-generalized metric space. We renamed it as* κ-*JS-generalized metric space* (*in short, a* $\kappa_{JS}$-*MS*).

**Remark 0.1** [22]. *If the set c*(J_d_, *z*, x) *is empty for every* x ∈ *z*, *then* (*z*, J_d_) *is a* $\kappa_{JS}$-*MS if and only if* (J_d1_) *and* (J_d2_) *are satisfied*.

Many examples of $\kappa_{JS}$-MS can be found in [22, 23, 26].

**Example 0.1** [22].

1. *A modular metric space* (*z*, ρ) is a $\rho_{JS}$-*MS*.
2. *A standard metric space is a* $1_{JS}$ -*MS*.
3. *A 2-metric space is a* $2_{JS}$ -*MS*.

**Definition 0.3**. [22] *Let* (*z*, J_d_) *be a* $\kappa_{JS}$-*MS and* x ∈ *z*.

1. *A sequence* {x_p_} ⊆ *z* *is said to be* J_d_-*convergent and* J_d_-*converges to* x *if* {x_p_} ∈ J_d_, *z*, x). *In such case, we will write* $\left\{x_{p}\right\}\overset{J_{d}}{\to }x.$
2. *a sequence* {x_p_} ⊆ *z* *is said to be* J_d_-*Cauchy if*
  $$\begin{array}{c} \underset{p,q\to \infty }{\text{lim}}\;J_{d}\left(x_{p},x_{p+q}\right)=0. \end{array}$$ (4)
3. *A* $\kappa_{JS}$ -*MS* (*z*, J_d_) *is said to be complete if every* J_d_-*Cauchy sequence in* *z* *is* J_d_-*convergent*.

**Proposition 0.1** [22]. *Let* (*z*, J_d_) *be a* $\kappa_{JS}$-*MS*, {x_p_} *be a sequence in* *z* *and* (x, y) ∈ *z* × *z*. *If* {x_p_} *is* J_d_-*convergent and* J_d_-*converges to* x *and* {x_p_} J_d_-*converges to* y, *then* x = y.

**Remark 0.2**. *Jleli and Samet defined* J_d_-*Cauchy sequence as*

$$\begin{array}{c} \underset{p,q\to \infty }{\text{lim}}\;J_{d}\left(x_{p},x_{q}\right)=0. \end{array}$$ (5)

*Clearly*, (5) *implies* (4), *the opposite, however, need not be true* [23]. *From here on, we assume that* J_d_-*Cauchy sequences are given by* (5).

**Definition 0.4** [23]. *Let* (*z*, J_d_) *be a* $\kappa_{JS}$-*MS and* ξ: *z* → *z*. *For* x_0_ ∈ *z*, *denote* δ(J_d_, ξ, x_0_), *the* J_d_-*diameter of the orbit of* x_0_ *by* ξ, $O_{\xi }\left(x_{0}\right)=\left\{\xi^{p}x_{0}:p\in N\right\}$, *and is defined as*,

$$\begin{array}{c} \delta \left(J_{d},\xi ,x_{0}\right)=\text{sup}\left(\left\{J_{d}\left(\xi^{p}x_{0},\xi^{q}x_{0}\right):q,p\in N\right\}\right). \end{array}$$ (6)

**Definition 0.5** [23]. *Let* ${∼}_{R}$ *be a binary relation on* $\kappa_{JS}$-*MS* (*z*, J_d_). *A sequence* {x_p_} ⊆ *z* *is* ${∼}_{R}$ -*non-decreasing if* x_p_ ${∼}_{R}$ x_p+1_ *for each* p∈N.

**Definition 0.6** [23]. *A* $\kappa_{JS}$-*MS* (*z*, J_d_) *is called* ${∼}_{R}$ -*non-decreasing complete if every* ${∼}_{R}$ -*non-decreasing and* J_d_-*Cauchy sequence is* J_d_-*convergent in z*.

**Remark 0.3**. *Notice that every complete* $\kappa_{JS}$-*MS is also* ${∼}_{R}$ -*non-decreasing complete. As evidenced by the case below, the contrary is untrue*.

**Example 0.2**. *Let z* = (0, 1] *furnished with the Euclidean metric* *m*(x, y) = |x − y| *for each* x, y ∈ *z*. *Define a binary relation* ${∼}_{R}$ *on* *z* *by*

$$x{∼}_{R}y\;\;\text{if}\;\;0<x\leq y\leq 1.$$

*Then* (*z*, *m*) *is* ${∼}_{R}$ -*non-decreasing complete, however, the metric space is not complete*.

**Definition 0.7** [23]. *Let* (*z*, J_d_) *be a* $\kappa_{JS}$-*MS. A mapping* ξ: *z* → *z* *is* ${∼}_{R}$ -*non-decreasing-continuous at* ν ∈ *z if* {ξx_p_} ∈ J_d_, *z*, *s*ν) *for each* ${∼}_{R}$ -*non-decreasing sequence* {x_p_} ∈ J_d_, *z*, ν). *A mapping* ξ *is* ${∼}_{R}$ -*non-decreasing-continuous if it is* ${∼}_{R}$ -*non-decreasing-continuous at every point of z*.

**Remark 0.4**. [23] *Every continuous mapping is also* ${∼}_{R}$ -*non-decreasing-continuous, while the reverse is generally false, as seen in Example 4.6 of* [23].

By getting inspiration from the work of Karapinar *et al.* [23], here, we prove fixed point theorems for ℑ-contractions in the context of JS-generalized metric space.

## 2 Fundamental results

Let (*z*, J_d_) be a $\kappa_{JS}$-MS and let ξ be a self-mapping on *z*. Throughout this section, we denote, for all x, y ∈ *z*,

$$Q_{\xi }^{J_{d}}\left(x,y\right)=\text{max}\left\{J_{d}\left(x,y\right),J_{d}\left(x,\xi x\right),J_{d}\left(y,\xi y\right),J_{d}\left(x,\xi y\right),J_{d}\left(y,\xi x\right)\right\}.$$

Following [23], define for given $p_{0}\in N$,

$$\begin{array}{c} \delta_{p_{0}}\left(J_{d},\xi ,x_{0}\right)=\text{sup}\left(\left\{J_{d}\left(\xi^{p}x_{0},\xi^{q}x_{0}\right):q,p\in N,q,p\geq p_{0}\right\}\right). \end{array}$$ (7)

and

$$\begin{array}{c} \delta \left(J_{d},\xi ,x_{0}\right)=\delta_{0}\left(J_{d},\xi ,x_{0}\right)=\text{sup}\left(\left\{J_{d}\left(\xi^{p}x_{0},\xi^{q}x_{0}\right):q,p\in N\right\}\right). \end{array}$$ (8)

By the symmetry of J_d_, we can alternatively express

$$\begin{array}{c} \delta_{p_{0}}\left(J_{d},\xi ,x_{0}\right)=\text{sup}\left(\left\{J_{d}\left(\xi^{p}x_{0},\xi^{q}x_{0}\right):q,p\in N,q\geq p\geq p_{0}\right\}\right). \end{array}$$ (9)

Notice that if q,p∈N satisfy q ≥ p, then

$$\begin{array}{c} \delta_{q}\left(J_{d},\xi ,x_{0}\right)\leq \delta_{p}\left(J_{d},\xi ,x_{0}\right)\leq \delta \left(J_{d},\xi ,x_{0}\right). \end{array}$$ (10)

**Lemma 1.1**. *Let* (*z*, J_d_) *be a* $\kappa_{JS}$-*MS with a preorder* ${∼}_{R}$ *and let* ξ: *z* → *z be an* ${∼}_{R}$ -*non-decreasing mapping. Let* x_0_ ∈ *z be a point such that* x_0_ *and* ξx_0_ *are* ${∼}_{R}$ -*comparable. Assume, there is a function* ℑ:(0,∞)→R *such that*

$$\begin{array}{c} \xi x\neq \xi y\;\;\text{implies}\;\;\lambda +ℑ\left(J_{d}\left(\xi x,\xi y\right)\right)\leq ℑ\left(Q_{\xi }^{J_{d}}\left(x,y\right)\right), \end{array}$$ (11)

*for* x, y ∈ *z satisfying* $\text{x}{∼}_{R}\text{y}$ *and* λ > 0. *Then* (11) *holds for each* $\text{x},\text{y}\in O_{\xi }({\text{x}}_{0})$.

*Proof.* Consider the Picard sequence ${\left\{x_{p}=\xi x_{p-1}=\xi^{p}x_{0}\right\}}_{p\in N}$ of ξ based on x_0_. Suppose that ${\text{x}}_{0}{∼}_{R}{\text{x}}_{1}$. As ξ is ${∼}_{R}$ -non-decreasing, then ${\text{x}}_{1}=\xi {\text{x}}_{0}{∼}_{R}\xi {\text{x}}_{1}={\text{x}}_{2}$. Repeating this argument, we get, ${\text{x}}_{\text{p}}{∼}_{R}{\text{x}}_{\text{p}+1}$ for every p∈N. Since ${∼}_{R}$ is a preorder, then ${\text{x}}_{\text{p}}{∼}_{R}{\text{x}}_{\text{q}}$ for all q,p∈N such that p ≤ q. Furthermore, as condition (11) is symmetric on x and y, then (11) holds for each x_p_ and x_q_ (being q,p∈N arbitrary), so it holds for each $\text{x},\text{y}\in O_{\xi }({\text{x}}_{0})$.

**Lemma 1.2**. *Let* (*z*, J_d_) *be a* $\kappa_{JS}$-*MS and let* ξ: *z* → *z* *be a mapping. Let* x_0_ ∈ *z* *be a point for which there exists* $p_{0}\in N$ *such that* $\delta_{p_{0}}\left(J_{d},\xi ,x\right)<\infty$. *Assume, there is a non-decreasing function* ℑ:(0,∞)→R *obeying*

$\left({ℑ}_{J_{d}}\right)$ : ℑ(sup M) = sup ℑ(M) *for all* M ⊂ (0, ∞) *with* sup M > 0

*and*

$$\begin{array}{c} \xi x\neq \xi y\;\;\text{implies}\;\;\lambda +ℑ\left(J_{d}\left(\xi x,\xi y\right)\right)\leq ℑ\left(Q_{\xi }^{J_{d}}\left(x,y\right)\right), \end{array}$$ (12)

*for all* $\text{x},\text{y}\in O_{\xi }({\text{x}}_{0})$ *and* λ > 0. *Then*

$$\begin{array}{c} \delta_{\ell +1}\left(J_{d},\xi ,x_{0}\right)\leq \delta_{\ell }\left(J_{d},\xi ,x_{0}\right)-\lambda ,\;\text{for}\;\text{all}\;\ell \in N,\ell \geq p_{0}. \end{array}$$

*In particular,*

$$\begin{array}{c} \delta_{p_{0}+\ell }\left(J_{d},\xi ,x_{0}\right)\leq \delta_{p_{0}}\left(J_{d},\xi ,x_{0}\right)-\ell \lambda ,\;\text{for}\;\text{all}\;\ell \in N,\ell \geq p_{0}. \end{array}$$

*Proof.* Let ℓ∈N be such that ℓ ≥ p_0_. From (10), we have

$$\begin{array}{c} \delta_{\ell +1}\left(J_{d},\xi ,x_{0}\right)\leq \delta_{\ell }\left(J_{d},\xi ,x_{0}\right)\leq \delta_{p_{0}}\left(J_{d},\xi ,x_{0}\right)<\infty . \end{array}$$

Let q,p∈N be such that p ≥ q ≥ ℓ + 1. Denote

$$\begin{array}{c} \Omega_{\ell }=\left\{J_{d}\left(\xi^{r}x_{0},\xi^{s}x_{0}\right):r,s\in N,\;\;r,s\geq \ell \right\}, \end{array}$$

then

$$\begin{array}{c} J_{d}\left(\xi^{p-1}x_{0},\xi^{q-1}x_{0}\right),J_{d}\left(\xi^{p-1}x_{0},\xi^{p}x_{0}\right),J_{d}\left(\xi^{q-1}x_{0},\xi^{q}x_{0}\right),J_{d}\left(\xi^{p-1}x_{0},\xi^{q}x_{0}\right),J_{d}\left(\xi^{q-1}x_{0},\xi^{p}x_{0}\right)\in \Omega_{\ell }. \end{array}$$

Hence

$$\begin{array}{cc} & \text{max}\{J_{d}\left(\xi^{p-1}x_{0},\xi^{q-1}x_{0}\right),J_{d}\left(\xi^{p-1}x_{0},\xi^{p}x_{0}\right),J_{d}\left(\xi^{q-1}x_{0},\xi^{q}x_{0}\right),J_{d}\left(\xi^{p-1}x_{0},\xi^{q}x_{0}\right),\\ & J_{d}\left(\xi^{q-1}x_{0},\xi^{p}x_{0}\right)\}\leq \text{sup}\Omega_{\ell }=\delta_{\ell }\left(J_{d},\xi ,x_{0}\right). \end{array}$$ (13)

From (13) and (13), we obtain

$$\begin{array}{cc} \lambda +ℑ(J_{d}\left(\xi^{p}x_{0},\xi^{q}x_{0}\right)) & \leq ℑ(Q_{\xi }^{J_{d}}\left(\xi^{p-1}x_{0},\xi^{q-1}x_{0}\right))\\ & \leq ℑ(\delta_{\ell }\left(J_{d},\xi ,x_{0}\right)). \end{array}$$ (14)

By the virtue of $\left({ℑ}_{J_{d}}\right)$ and (14), we get

$$\begin{array}{cc} ℑ(\delta_{\ell +1}\left(J_{d},\xi ,x_{0}\right)) & =ℑ(\text{sup}\left\{J_{d}\left(\xi^{p}x_{0},\xi^{q}x_{0}\right):q,p\in N,\;\ell +1\leq n\leq p\right\})\\ & =\text{sup}\{ℑ\left(J_{d}\left(\xi^{p}x_{0},\xi^{q}x_{0}\right)\right):q,p\in N,\;\ell +1\leq n\leq p\}\\ & \leq \text{sup}\{ℑ\left(\delta_{\ell }\left(J_{d},\xi ,x_{0}\right)\right)-\lambda :q,p\in N,\;\ell +1\leq n\leq p\}\\ & \leq ℑ(\delta_{\ell }\left(J_{d},\xi ,x_{0}\right))-\lambda . \end{array}$$ (15)

Continuing this argument and recognizing that ℑ is non-decreasing, we get for all ℓ∈N

$$\begin{array}{cc} ℑ(\delta_{p_{0}+\ell }\left(J_{d},\xi ,x_{0}\right)) & \leq ℑ(\delta_{p_{0}+\ell -1}\left(J_{d},\xi ,x_{0}\right))-\lambda \\ & \leq ℑ(\delta_{p_{0}+\ell -2}\left(J_{d},\xi ,x_{0}\right))-2\lambda \\ & \vdots \\ & \leq ℑ(\delta_{p_{0}}\left(J_{d},\xi ,x_{0}\right))-\ell \lambda . \end{array}$$

## 3 Main results

**THEOREM 2.1**. *Let* (*z*, J_d_) *be a* $\kappa_{JS}$-*MS with a preorder* ${∼}_{R}$ *and let* ξ: *z* → *z* *be an* ${∼}_{R}$ -*non-decreasing mapping. Let* x_0_ ∈ *z* *be a point such that* ${\text{x}}_{0}{∼}_{R}\xi {\text{x}}_{0}$ *and* $\delta_{p_{0}}\left(J_{d},\xi ,x\right)<\infty$ *for some* $p_{0}\in N$. *Assume, there is a non-decreasing function* ℑ:(0,∞)→R *obeying* $\left({ℑ}_{2}^{\prime }\right)$, $\left({ℑ}_{J_{d}}\right)$ *and* (11) *for all* $\text{x},\text{y}\in O_{\xi }({\text{x}}_{0})$ *and* λ > 0. *Then the sequence* ${\left\{\xi^{p}x_{0}\right\}}_{p\in N}$ *based on* x_0_ *is* ${∼}_{R}$ -*non-decreasing and* J_d_-*Cauchy sequence*.

*Furthermore, if* (*z*, J_d_) *is* ${∼}_{R}$ -*non-decreasing-complete, then* ${\left\{\xi^{p}x_{0}\right\}}_{p\in N}$ J_d_-*converges to a point* ν ∈ *z that obeys*

$$\begin{array}{c} J_{d}\left(\nu ,\nu \right)=0. \end{array}$$

*Additionally, if* ξ *is* ${∼}_{R}$ -*non-decreasing-continuous, then* ν = ξν.

*Proof.* Consider the Picard sequence ${\left\{x_{p}=\xi x_{p-1}=\xi^{p}x_{0}\right\}}_{p\in N}$ of ξ based on x_0_. As shown in the proof of Lemma 1.1, ${\left\{\xi^{p}x_{0}\right\}}_{p\in N}$ is ${∼}_{R}$ -non-decreasing. If ξ^p^x_0_ = ξ^q^x_0_, then J_d_(x_p_, x_q_) = 0 for every q, p ≥ p_0_. In particular,

$$\begin{array}{c} \underset{p,q\to \infty }{\text{lim}}\;J_{d}\left(x_{p},x_{q}\right)=0. \end{array}$$

Consider ξ^p^x_0_ ≠ ξ^q^x_0_ and $q,p\in N,p_{0}\leq p<q$ for some $p_{0}\in N$, then by using Lemma 1.2, we have

$$\begin{array}{c} ℑ\left(J_{d}\left(\xi^{p}x_{0},\xi^{q}x_{0}\right)\right)\leq ℑ\left(\delta_{q+p}\left(J_{d},\xi ,x_{0}\right)\right)\leq ℑ\left(\delta_{q}\left(J_{d},\xi ,x_{0}\right)\right)-p\lambda . \end{array}$$ (16)

Letting limit in (16) as p, q → ∞, we have

$$\begin{array}{c} \underset{p,q\to \infty }{\text{lim}}\;ℑ\left(J_{d}\left(\xi^{p}x_{0},\xi^{q}x_{0}\right)\right)=\infty . \end{array}$$ (17)

Taking into account of $\left({ℑ}_{2}^{\prime }\right)$, we have

$$\begin{array}{c} \underset{p,q\to \infty }{\text{lim}}\;J_{d}\left(\xi^{p}x_{0},\xi^{q}x_{0}\right)=0. \end{array}$$

Hence ${\left\{\xi^{p}x_{0}\right\}}_{p\in N}$ J_d_-Cauchy sequence. Since (*z*, J_d_) is ${∼}_{R}$ -non-decreasing-complete, there exists ν ∈ *z* such that $\left\{x_{p}\right\}\overset{J_{d}}{\to }\nu$. By using (J_d3_), we get

$$\begin{array}{c} 0\leq J_{d}\left(\nu ,\nu \right)\leq \kappa \underset{p\to \infty }{\text{lim}}\;\text{sup}\;J_{d}\left(x_{p},\nu \right)=0, \end{array}$$ (18)

which implies J_d_(ν, ν) = 0.

Moreover, as we additionally assume that ξ is ${∼}_{R}$ -non-decreasing-continuous, then

$$\begin{array}{c} \left\{x_{p+1}=\xi^{p}x_{0}=\xi x_{p}\right\}\overset{J_{d}}{\to }\xi \nu . \end{array}$$

Proposition 0.1 guarantees that ξν = ν, so ν is a fixed point of ξ.

**Example 2.1**. *Consider the function* ℑ:(0,∞)→R *defined as*

$$ℑ\left(t\right)=t^{t}\;\text{for}\;\text{all}\;t\in \left(0,\infty \right).$$

*Then* ℑ *is non-decreasing and continuous but does not satisfies* $\left({ℑ}_{2}^{\prime }\right)$.

So, in next theorem, we replace the condition of $\left({ℑ}_{2}^{\prime }\right)$ by the continuity of ℑ in Theorem 2.1 and we denote by W, the collection of all functions ℑ:(0,∞)→R that are continuous and non-decreasing.

**THEOREM 2.2**. *Let* (*z*, J_d_) *be a* $\kappa_{JS}$-*MS with a preorder* ${∼}_{R}$ *and let* ξ: *z* → *z* *be an* ${∼}_{R}$ -*non-decreasing self-mapping. Let* x_0_ ∈ *z be a point such that* ${\text{x}}_{0}{∼}_{R}\xi {\text{x}}_{0}$, $\delta_{p_{0}}\left(J_{d},\xi ,x\right)<\infty$ *for some* $p_{0}\in N$ *and the following holds true*:

$$\begin{array}{c} \underset{p_{0}+\ell \to \infty }{\text{lim}}\;\delta_{p_{0}+\ell }\left(J_{d},\xi ,x_{0}\right)=\underset{p_{0}\to \infty }{\text{lim}}\;\delta_{p_{0}}\left(J_{d},\xi ,x_{0}\right)\;\text{for}\;\text{arbitrary}\;\;\ell \in N\;\;\text{such}\;\text{that}\;\;\ell \geq p_{0}. \end{array}$$

*If there exists a function* ℑ∈W *and* λ > 0 *such that inequality* (11) *holds for all* $\text{x},\text{y}\in O_{\xi }({\text{x}}_{0})$, *then the sequence* ${\left\{\xi^{p}x_{0}\right\}}_{p\in N}$ *based on* x_0_ *is* ${∼}_{R}$ -*non-decreasing and* J_d_-*Cauchy sequence*.

*Furthermore, if* (*z*, J_d_) *is* ${∼}_{R}$ -*non-decreasing-complete, then* ${\left\{\xi^{p}x_{0}\right\}}_{p\in N}$ J_d_-*converges to a point* ν ∈ *z* *that satisfies*

$$\begin{array}{c} J_{d}\left(\nu ,\nu \right)=0. \end{array}$$

*Additionally, if* ξ *is* ${∼}_{R}$ -*non-decreasing-continuous, then* ν = ξν.

*Proof.* Consider the Picard sequence ${\left\{x_{p}=\xi x_{p-1}=\xi^{p}x_{0}\right\}}_{p\in N}$ of ξ based on x_0_. As shown in the proof of Lemma 1.1, ${\left\{\xi^{p}x_{0}\right\}}_{p\in N}$ is ${∼}_{R}$ -non-decreasing. If $\delta_{p_{0}}\left(J_{d},\xi ,x_{0}\right)=0$, then J_d_(x_p_, x_q_) = 0 for all q, p ≥ p_0_. In particular,

$$\begin{array}{c} \underset{q,p\to \infty }{\text{lim}}\;J_{d}\left(x_{p},x_{q}\right)=0. \end{array}$$

Consider $\delta_{p_{0}}\left(J_{d},\xi ,x_{0}\right)>0$ and $q,p\in N,p_{0}\leq p<q$ for some $p_{0}\in N$. Denote

$$\begin{array}{c} \Omega_{p_{0}}=\left\{J_{d}\left(\xi^{r}x_{0},\xi^{s}x_{0}\right):r,s\in N,\;\;r,s\geq p_{0}\right\}, \end{array}$$

then

$$\begin{array}{c} J_{d}\left(\xi^{p-1}x_{0},\xi^{q-1}x_{0}\right),J_{d}\left(\xi^{p-1}x_{0},\xi^{p}x_{0}\right),J_{d}\left(\xi^{q-1}x_{0},\xi^{q}x_{0}\right),J_{d}\left(\xi^{p-1}x_{0},\xi^{q}x_{0}\right),J_{d}\left(\xi^{q-1}x_{0},\xi^{p}x_{0}\right)\in \Omega_{p_{0}}. \end{array}$$

Hence

$$\begin{array}{c} \text{max}\{J_{d}\left(\xi^{p-1}x_{0},\xi^{q-1}x_{0}\right),J_{d}\left(\xi^{p-1}x_{0},\xi^{p}x_{0}\right),J_{d}\left(\xi^{q-1}x_{0},\xi^{q}x_{0}\right),J_{d}\left(\xi^{p-1}x_{0},\xi^{q}x_{0}\right),\\ J_{d}\left(\xi^{q-1}x_{0},\xi^{p}x_{0}\right)\}\leq \text{sup}\Omega_{p_{0}}=\delta_{p_{0}}\left(J_{d},\xi ,x_{0}\right). \end{array}$$ (19)

Assume that ${\text{lim}}_{p_{0}+\ell \to \infty }\delta_{p_{0}+\ell }\left(J_{d},\xi ,x_{0}\right)={\text{lim}}_{p_{0}\to \infty }\delta_{p_{0}}\left(J_{d},\xi ,x_{0}\right)=℧$ for ℓ∈N be an arbitrary such that ℓ ≥ p_0_. Then, by using inequalities (11) and (19) and continuity of ℑ, we obtain

$$\begin{array}{cc} \lambda +ℑ(℧) & =\lambda +ℑ(\underset{p_{0}+\ell \to \infty }{\text{lim}}\;\delta_{p_{0}+\ell }\left(J_{d},\xi ,x_{0}\right))\\ & =\lambda +ℑ(\underset{p,q\to \infty }{\text{lim}\;\text{sup}}\;J_{d}\left(\xi^{p}x_{0},\xi^{q}x_{0}\right))\\ & =\underset{p,q\to \infty }{\text{lim}\;\text{sup}}\;(\lambda +ℑ(J_{d}\left(\xi^{p}x_{0},\xi^{q}x_{0}\right)))\\ & \leq \underset{p,q\to \infty }{\text{lim}\;\text{sup}}\;(ℑ(\delta_{p_{0}}\left(J_{d},\xi ,x_{0}\right)))\\ & =ℑ(\underset{p_{0}\to \infty }{\text{lim}}\;\delta_{p_{0}}\left(J_{d},\xi ,x_{0}\right))\\ & =ℑ(℧), \end{array}$$ (20)

a contradiction because λ > 0. Hence ${\left\{\xi^{p}x_{0}\right\}}_{p\in N}$ J_d_-Cauchy sequence. Since (*z*, J_d_) is ${∼}_{R}$ -non-decreasing-complete, there is ν ∈ *z* such that $\left\{x_{p}\right\}\overset{J_{d}}{\to }\nu$. By using (J_d3_), we get

$$\begin{array}{c} 0\leq J_{d}\left(\nu ,\nu \right)\leq \kappa \;\underset{p\to \infty }{\text{lim}}\;\text{sup}\;J_{d}\left(x_{p},\nu \right)=0, \end{array}$$ (21)

which implies J_d_(ν, ν) = 0.

Moreover, as we additionally assume that ξ is ${∼}_{R}$ -non-decreasing-continuous, then

$$\begin{array}{c} \left\{x_{p+1}=\xi^{p}x_{0}=\xi x_{p}\right\}\overset{J_{d}}{\to }\xi \nu . \end{array}$$

Proposition 0.1 guarantees that ξν = ν.

**Example 2.2**. *Let z* = [0, 1] ∪ {2} *and let* J_d_: *z* × *z* → [0, ∞] *be a function defined by*

$$J_{d}\left(x,y\right)=\{\begin{array}{cc} 10 & \text{if}\;\text{either}\;\;\;(x,y)=(0,2)\;\;\;(x,y)=(2,0),\\ \left|x-y\right| & \text{otherwise}. \end{array}$$

*Then* (*z*, J_d_) *is complete* $\kappa_{JS}$-*MS* (*see* [23]. *Define a binary relation* ${∼}_{R}$ *on z by*

$$x{∼}_{R}y\;if\;0<x\leq y\leq 1,$$

*then* ${∼}_{R}$ *is a preorder and* (*z*, J_d_) *is a preordered space. Let* x_0_ = 0.25 ∈ *z be a point such that* 0 < x_0_ = 0.25 < 1 = ξ(0.25) = ξx_0_, *then* ${\text{x}}_{0}{∼}_{R}\xi {\text{x}}_{0}$ *and*

$$O_{\xi }\left(0.25\right)=\left\{0.25,1,0.5,0.5,0.5,\cdots \right\}.$$

*Also*, $\delta_{p_{0}}\left(J_{d},\xi ,x\right)=0.5<\infty$ *and* ${\text{lim}}_{p_{0}+\ell \to \infty }\delta_{p_{0}+\ell }\left(J_{d},\xi ,x_{0}\right)={\text{lim}}_{p_{0}\to \infty }\delta_{p_{0}}\left(J_{d},\xi ,x_{0}\right)$ *for any* $p_{0},\ell \in N$ *such that* ℓ ≥ p_0_. *Now Define* ξ: *z* → *z and* ℑ: (0, ∞) → (−∞, ∞) *by*

$$\xi \left(x\right)=\{\begin{array}{cc} 0.5 & \text{if}\;\;\;x\in \{0.5,1\},\\ 1 & \text{if}\;\;\;x=0.25,\\ 0 & \text{otherwise} \end{array}\;\text{and}\;ℑ\left(r\right)=\text{ln}\;r\;\;\;\text{for}\;\text{all}\;\;\;r\in \left(0,\infty \right)$$

*respectively, then* ℑ *is continuous and non-decreasing function. Let* $\text{x},\text{y}\in O_{\xi }(0.25)$ *such that* ξx ≠ ξy, *then there arises two cases*:

**Case 1:** *When* x = 0.25, y = 0.5, *then there exists* λ = 0.25 > 0 *such that*

$$\begin{array}{c} \;ℑ\left(J_{d}\left(\xi x,\xi y\right)\right)-ℑ\left(Q_{\xi }^{J_{d}}\left(x,y\right)\right)\\ =ℑ\left(J_{d}\left(\xi 0.25,\xi 0.5\right)\right)-ℑ\left(Q_{\xi }^{J_{d}}\left(0.25,0.5\right)\right)\\ =ℑ\left(J_{d}\left(1,0.5\right)\right)-ℑ\left(\text{max}\left\{J_{d}\left(0.25,0.5\right),J_{d}\left(0.25,1\right),J_{d}\left(0.5,0.5\right),J_{d}\left(0.25,0.5\right),J_{d}\left(0.5,1\right)\right\}\right)\\ =ℑ(0.5)-ℑ(\text{max}\{0.25,0.75,0,0.25,0.5\})\\ =ℑ(0.5)-ℑ(0.75)=\text{ln}\;(0.5)-\text{ln}\;(0.75)\\ =-0.4055<-0.25=-\lambda . \end{array}$$

**Case 2:** *When* x = 0.25, y = 1, *then there exists* λ = 0.25 > 0 *such that*

$$\begin{array}{c} \;ℑ\left(J_{d}\left(\xi x,\xi y\right)\right)-ℑ\left(Q_{\xi }^{J_{d}}\left(x,y\right)\right)\\ =ℑ\left(J_{d}\left(\xi 0.25,\xi 1\right)\right)-ℑ\left(Q_{\xi }^{J_{d}}\left(0.25,1\right)\right)\\ =ℑ\left(J_{d}\left(1,0.5\right)\right)-ℑ\left(\text{max}\left\{J_{d}\left(0.25,1\right),J_{d}\left(0.25,1\right),J_{d}\left(1,0.5\right),J_{d}\left(0.25,0.5\right),J_{d}\left(1,1\right)\right\}\right)\\ =ℑ(0.5)-ℑ(\text{max}\{0.75,0.75,0.5,0.25,0\})\\ =ℑ(0.5)-ℑ(0.75)=\text{ln}\;(0.5)-\text{ln}\;(0.75)\\ =-0.4055<-0.25=-\lambda . \end{array}$$

*This show that* ℑ *satisfies* (11) *for all* $\text{x},\text{y}\in O_{\xi }({\text{x}}_{0})$. *Thus, all hypotheses of Theorem 2.2 hold true and* {0, 0.5} *is the set of all fixed points of* ξ.

## 4 Consequences

In this section, we find more results involving stronger contractive conditions.

**Corollary 3.1**. *Let* (*z*, J_d_) *be a* $\kappa_{JS}$-*MS with a preorder* ${∼}_{R}$ *and let* ξ: *z* → *z be an* ${∼}_{R}$ -*non-decreasing mapping. Let* x_0_ ∈ *z be a point such that* ${\text{x}}_{0}{∼}_{R}\xi {\text{x}}_{0}$ *and* $\delta_{p_{0}}\left(J_{d},\xi ,x\right)<\infty$ *for some* $p_{0}\in N$. *Assume there is b* ∈ (0, 1) *such that*

$$\begin{array}{c} J_{d}\left(\xi x,\xi y\right)\leq bQ_{\xi }^{J_{d}}\left(x,y\right), \end{array}$$ (22)

*for all* $\text{x},\text{y}\in O_{\xi }({\text{x}}_{0})$. *Then the sequence* ${\left\{\xi^{p}x_{0}\right\}}_{p\in N}$ *based on* x_0_ *is* ${∼}_{R}$ -*non-decreasing and* J_d_-*Cauchy sequence*.

*Furthermore, if* (*z*, J_d_) *is* ${∼}_{R}$ -*non-decreasing-complete, then* ${\left\{\xi^{p}x_{0}\right\}}_{p\in N}$ J_d_-*converges to a point* ν ∈ *z that meets*

$$\begin{array}{c} J_{d}\left(\nu ,\nu \right)=0. \end{array}$$

*Additionally, if* ξ *is* ${∼}_{R}$ -*non-decreasing-continuous, then* ν = ξν.

*Proof.* Define ℑ:(0,∞)→R by ℑ(s) = ln s for all s ∈ (0, ∞). Put $b=\frac{1}{e^{\lambda }}$. Inequality (26) implies (11). Hence, all of the requirements of Theorem 2.1 have been met, and the proof is concluded.

**Corollary 3.2**. *Let* (*z*, J_d_) *be a* $\kappa_{JS}$-*MS with a preorder* ${∼}_{R}$ *and let* ξ: *z* → *z* *be an* ${∼}_{R}$ -*non-decreasing mapping. Let* x_0_ ∈ *z be a point such that* ${\text{x}}_{0}{∼}_{R}\xi {\text{x}}_{0}$ *and* $\delta_{p_{0}}\left(J_{d},\xi ,x\right)<\infty$ *for some* $p_{0}\in N$. *Assume, there is a non-decreasing function* ℑ: (0, ∞) → (−∞, ∞) *fulfilling* $\left({ℑ}_{2}^{\prime }\right)$, $\left({ℑ}_{J_{d}}\right)$

$$\begin{array}{c} \xi x\neq \xi y\;\;\text{implies}\;\;\lambda +ℑ\left(J_{d}\left(\xi x,\xi y\right)\right)\leq ℑ\left(Q_{\xi }^{J_{d}}\left(x,y\right)\right),\;\;\text{for}\;\text{all}\;\;x,y\in z\;\;\text{such}\;\text{that}\;\;x{∼}_{R}y, \end{array}$$ (23)

*for* λ > 0. *Then the sequence* ${\left\{\xi^{p}x_{0}\right\}}_{p\in N}$ *based on* x_0_ *is* ${∼}_{R}$ -*non-decreasing and* J_d_-*Cauchy sequence*.

*Furthermore, if* (*z*, J_d_) *is* ${∼}_{R}$ -*non-decreasing-complete, then* ${\left\{\xi^{p}x_{0}\right\}}_{p\in N}$ J_d_-*converges to a point* ν ∈ *z* *that meets*

$$\begin{array}{c} J_{d}\left(\nu ,\nu \right)=0. \end{array}$$

*Additionally, if* ξ *is* ${∼}_{R}$ -*non-decreasing-continuous, then* ν = ξν.

*Proof.* Let the contractivity condition (27) hold for all x, y ∈ *z* such that $\text{x}{∼}_{R}\text{y}$, then Lemma 1.1 guarantees that it also holds for $\text{x},\text{y}\in O_{\xi }({\text{x}}_{0})$. So due to Theorem 2.1, we obtained the result.

**Corollary 3.3**. *Let* (*z*, J_d_) *be a* $\kappa_{JS}$-*MS with a preorder* ${∼}_{R}$ *and let* ξ: *z* → *z be an* ${∼}_{R}$ -*non-decreasing mapping. Let* x_0_ ∈ *z be a point such that* ${\text{x}}_{0}{∼}_{R}\xi {\text{x}}_{0}$, $\delta_{p_{0}}\left(J_{d},\xi ,x\right)<\infty$ *for some* $p_{0}\in N$ *and the following holds true*:

$$\begin{array}{c} \underset{p_{0}+\ell \to \infty }{\text{lim}}\;\delta_{p_{0}+\ell }\left(J_{d},\xi ,x_{0}\right)=\underset{p_{0}\to \infty }{\text{lim}}\;\delta_{p_{0}}\left(J_{d},\xi ,x_{0}\right)\;\text{for}\;\text{arbitrary}\;\;\ell \in N\;\;\text{such}\;\text{that}\;\;\ell \geq p_{0}. \end{array}$$

*If there exists a function* ℑ∈W *and* λ > 0 *fulfilling the inequality* (27), *then the sequence* ${\left\{\xi^{p}x_{0}\right\}}_{p\in N}$ *based on* x_0_ *is* ${∼}_{R}$ -*non-decreasing and* J_d_-*Cauchy sequence*.

*Furthermore, if* (*z*, J_d_) *is* ${∼}_{R}$ -*non-decreasing-complete, then* ${\left\{\xi^{p}x_{0}\right\}}_{p\in N}$ J_d_-*converges to a point* ν ∈ *z that meets*

$$\begin{array}{c} J_{d}\left(\nu ,\nu \right)=0. \end{array}$$

*Additionally, if* ξ *is* ${∼}_{R}$ -*non-decreasing-continuous, then* ν = ξν.

*Proof.* By using the same reason as in the proof of Corollary 3.2, Theorem 2.2 gives the result.

**Corollary 3.4**. *Let* (*z*, J_d_) *be a* $\kappa_{JS}$-*MS with a partial order* ≪ *and let* ξ: *z* → *z be an* ≪-*non-decreasing mapping. Let* x_0_ ∈ *z be a point such that* x_0_ ≪ ξx_0_ *and* $\delta_{p_{0}}\left(J_{d},\xi ,x\right)<\infty$ *for some* $p_{0}\in N$. *Assume, there is a non-decreasing function* ℑ: (0, ∞) → (−∞, ∞) *satisfying* $\left({ℑ}_{2}^{\prime }\right)$, $\left({ℑ}_{J_{d}}\right)$ *and* (11) *for all* $\text{x},\text{y}\in O_{\xi }({\text{x}}_{0})$ *and* λ > 0. *Then, the sequence* ${\left\{\xi^{p}x_{0}\right\}}_{p\in N}$ *based on* x_0_ *is* ≪-*non-decreasing and* J_d_-*Cauchy sequence*.

*Furthermore, if* (*z*, J_d_) *is* ≪-*non-decreasing-complete, then* ${\left\{\xi^{p}x_{0}\right\}}_{p\in N}$ J_d_-*converges to a point* ν ∈ *z* *that meets*

$$\begin{array}{c} J_{d}\left(\nu ,\nu \right)=0. \end{array}$$

*Additionally, if* ξ *is* ≪-*non-decreasing-continuous, then* ν = ξν.

*Proof.* Due to the fact that a partial order ≪ is a preorder ${∼}_{R}$, the conclusion is reached by applying Theorem ref 2.1.

**Corollary 3.5**. *Let* (*z*, J_d_) *be a* $\kappa_{JS}$-*MS with a partial order* ≪ *and let* ξ: *z* → *z be an* ≪-*non-decreasing mapping. Let* x_0_ ∈ *z be a point such that* x_0_ ≪ ξx_0_, $\delta_{p_{0}}\left(J_{d},\xi ,x\right)<\infty$ *for some* $p_{0}\in N$ *and the following holds true*:

$$\begin{array}{c} \underset{p_{0}+\ell \to \infty }{\text{lim}}\;\delta_{p_{0}+\ell }\left(J_{d},\xi ,x_{0}\right)=\underset{p_{0}\to \infty }{\text{lim}}\;\delta_{p_{0}}\left(J_{d},\xi ,x_{0}\right)\;\text{for}\;\text{arbitrary}\;\;\ell \in N\;\;\text{such}\;\text{that}\;\;\ell \geq p_{0}. \end{array}$$

*If there exists a function* ℑ∈W *and* λ > 0 *fulfilling the inequality* (11) *for all* $\text{x},\text{y}\in O_{\xi }({\text{x}}_{0})$, *then the sequence* ${\left\{\xi^{p}x_{0}\right\}}_{p\in N}$ *based on* x_0_ *is* ≪-*non-decreasing and* J_d_-*Cauchy sequence*.

*Furthermore, if* (*z*, J_d_) *is* ≪-*non-decreasing-complete, then* ${\left\{\xi^{p}x_{0}\right\}}_{p\in N}$ J_d_-*converges to a point* ν ∈ *z* *that meets*

$$\begin{array}{c} J_{d}\left(\nu ,\nu \right)=0. \end{array}$$

*Additionally, if* ξ *is* ≪-*non-decreasing-continuous, then* ν = ξν.

*Proof.* Due to the fact that a partial order ≪ is a preorder ${∼}_{R}$, the conclusion is reached by applying Theorem ref 2.2.

**Corollary 3.6**. *Let* (*z*, J_d_) *be a* $\kappa_{JS}$-*MS with a preorder* ${∼}_{R}$ *and let* ξ: *z* → *z be an* ${∼}_{R}$ -*non-decreasing mapping. Let* x_0_ ∈ *z be a point such that* ${\text{x}}_{0}{∼}_{R}\xi {\text{x}}_{0}$ *and* $\delta_{p_{0}}\left(J_{d},\xi ,x\right)<\infty$ *for some* $p_{0}\in N$. *Assume, there is a non-decreasing function* ℑ: (0, ∞) → (−∞, ∞) *satisfying* $\left({ℑ}_{2}^{\prime }\right)$, $\left({ℑ}_{J_{d}}\right)$ *and*

$$\begin{array}{c} \lambda +ℑ\left(J_{d}\left(\xi x,\xi y\right)\right)\leq ℑ(\text{max}\{J_{d}\left(x,y\right),\frac{J_{d}\left(x,\xi x\right)+J_{d}\left(y,\xi y\right)}{2},\frac{J_{d}\left(x,\xi y\right)+J_{d}\left(y,\xi x\right)}{2}\}), \end{array}$$ (24)

*for all* $\text{x},\text{y}\in O_{\xi }({\text{x}}_{0})$ *with* ξx ≠ ξy *and* λ > 0. *Then the sequence* ${\left\{\xi^{p}x_{0}\right\}}_{p\in N}$ *based on* x_0_ *is* ${∼}_{R}$ -*non-decreasing and* J_d_-*Cauchy sequence*.

*Furthermore, if* (*z*, J_d_) *is* ${∼}_{R}$ -*non-decreasing-complete, then* ${\left\{\xi^{p}x_{0}\right\}}_{p\in N}$ J_d_-*converges to a point* ν ∈ *z that satisfies*

$$\begin{array}{c} J_{d}\left(\nu ,\nu \right)=0. \end{array}$$

*Additionally, if* ξ *is* ${∼}_{R}$ -*non-decreasing-continuous, then* ν = ξν.

*Proof.* Since $\frac{\left(r+s\right)}{2}\leq \text{max}\left\{s,r\right\}$ for all s, r ∈ [0, ∞], so inequality (28) implies inequality (11) and Theorem 2.1 leads to the conclusion.

**Corollary 3.7**. *Let* (*z*, J_d_) *be a* $\kappa_{JS}$-*MS with a preorder* ${∼}_{R}$ *and let* ξ: *z* → *z be an* ${∼}_{R}$ -*non-decreasing mapping. Let* x_0_ ∈ *z be a point such that* ${\text{x}}_{0}{∼}_{R}\xi {\text{x}}_{0}$ *and* $\delta_{p_{0}}\left(J_{d},\xi ,x\right)<\infty$ *for some* $p_{0}\in N$. *Assume, there is a continuous non-decreasing function* ℑ: (0, ∞) → (−∞, ∞) *obeying*

$$\begin{array}{c} \lambda +ℑ\left(J_{d}\left(\xi x,\xi y\right)\right)\leq ℑ(\text{max}\{J_{d}\left(x,y\right),J_{d}\left(x,\xi x\right),J_{d}\left(y,\xi y\right),\frac{J_{d}\left(x,\xi y\right)+J_{d}\left(y,\xi x\right)}{2}\}), \end{array}$$ (25)

*for all* $\text{x},\text{y}\in O_{\xi }({\text{x}}_{0})$ *with* ξx ≠ ξy, λ > 0 *and the following*:

$$\begin{array}{c} \underset{p_{0}+\ell \to \infty }{\text{lim}}\;\delta_{p_{0}+\ell }\left(J_{d},\xi ,x_{0}\right)=\underset{p_{0}\to \infty }{\text{lim}}\;\delta_{p_{0}}\left(J_{d},\xi ,x_{0}\right)\;\text{for}\;\text{arbitrary}\;\;\ell \in N\;\;\text{such}\;\text{that}\;\;\ell \geq p_{0}. \end{array}$$

*Then the sequence* ${\left\{\xi^{p}x_{0}\right\}}_{p\in N}$ *based on* x_0_ *is* ${∼}_{R}$ -*non-decreasing and* J_d_-*Cauchy sequence*.

*Furthermore, if* (*z*, J_d_) *is* ${∼}_{R}$ -*non-decreasing-complete, then* ${\left\{\xi^{p}x_{0}\right\}}_{p\in N}$ J_d_-*converges to a point* ν ∈ *z that obeys*

$$\begin{array}{c} J_{d}\left(\nu ,\nu \right)=0. \end{array}$$

*Additionally, if* ξ *is* ${∼}_{R}$ -*non-decreasing-continuous, then* ν = ξν.

*Proof.* Since $\frac{\left(r+s\right)}{2}\leq \text{max}\left\{s,r\right\}$ for all s, r ∈ [0, ∞], so inequality (28) implies inequality (11) and the result follows from Theorem 2.2.

**Remark 3.1**. *Theorem 2.1, Theorem 2.2 and Corollaries 3.1-3.7 remain true if we do one or more of the following changes in their statement*:

- *exchange the* ${∼}_{R}$ -*non-decreasing-completeness of* (*z*, J_d_) *by the completeness of* (*z*, J_d_);
- *exchange the* ${∼}_{R}$ -*non-decreasing-continuity of* ξ *by continuity*;
- *exchange the preorder* ${∼}_{R}$ *by the trivial preorder* ${∼}_{Rz}$ *given by* $\text{x}{∼}_{Rz}\text{y}$ *for all* x, y ∈ *z*;
- *exchange, in the contractivity condition, for all* $\text{x},\text{y}\in O_{\xi }({\text{x}}_{0})$ *by for all* x, y ∈ *z* *such that* $\text{x}{∼}_{R}\text{y}$;
- *exchange the* $\kappa_{JS}$ -*MS by any of the abstract metric spaces that could be considered as a* $\kappa_{JS}$-*MS*: *b-metric spaces, modular spaces, and standard metric spaces*.
- *exchange the contractivity condition* (11) *by*
$$\begin{array}{c} \lambda +ℑ\left(J_{d}\left(\xi x,\xi y\right)\right)\leq ℑ(J_{d}\left(x,y\right)), \end{array}$$
  *for every* $\text{x},\text{y}\in O_{\xi }({\text{x}}_{0})$ *with* ξx ≠ ξy.

## 5 Existence of solution to RLC circuit’s current differential equation

**Fig 1:**
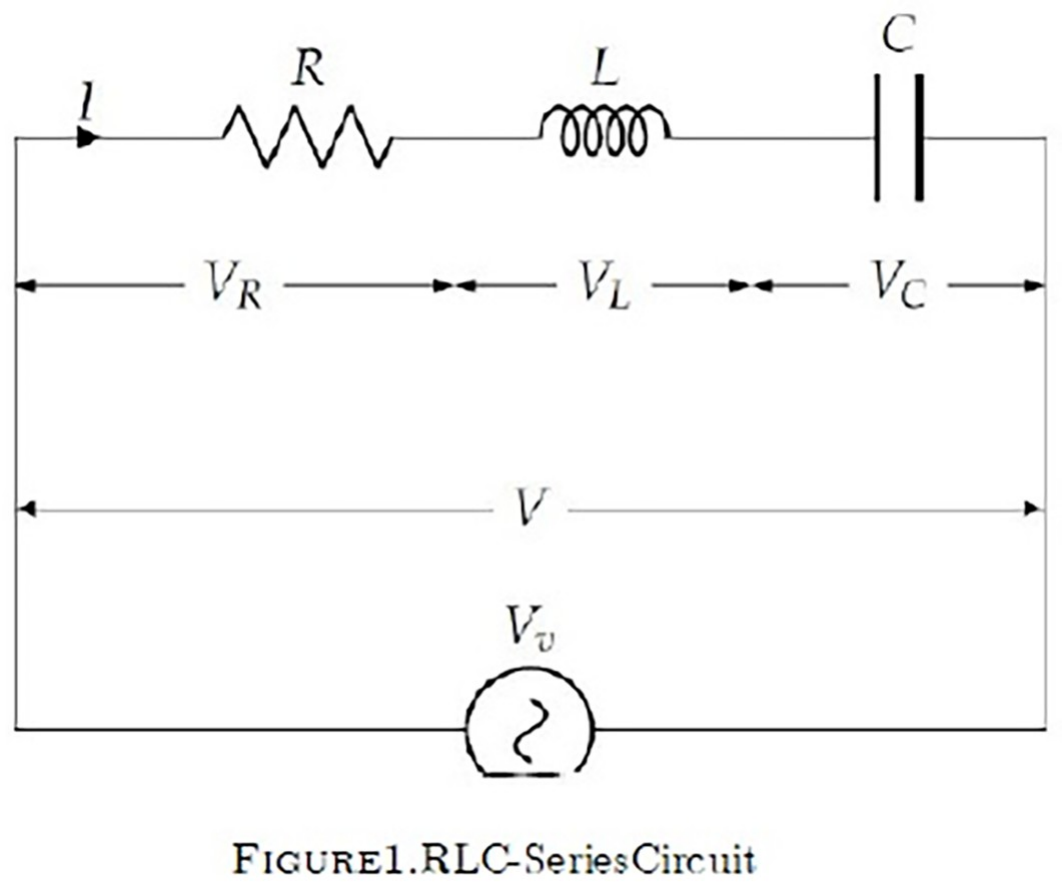
RLC series circuit.

A tuning circuit is a mathematical representation of the electric current in an RLC parallel circuit to present with a rudimentary knowledge of how light is converted into electricity. Consider the following series of electric circuit (Fig 1), which includes a resistor R, a capacitor C, an inductor L, a voltage V, and an electromotive force E. With the aid of Kirchhoff’s Voltage Law, related problems are mathematically modelled as initial value problems for second order ordinary differential equations of the form:

$$\{\begin{array}{l} \text{L}\frac{{\text{d}}^{2}q}{{\text{dt}}^{2}}+\text{R}\frac{\text{d}q}{\text{dt}}+\frac{q}{\text{C}}={\text{V}}_{\nu }(\text{t})\\ q(0)=q^{\prime }(0)=0, \end{array}$$ (26)

where V_ν_(t) = V.

In this part, we demonstrate the existence of the solution to the RLC differential equation (26). The problem (26) is identical to the following integral equation (see [29, 30]:

$$\begin{array}{c} q\left(t\right)=\int_{0}^{t}G\left(t,g\right)\hbar \left(g,q\left(g\right)\right)dg,\;t\in \left[0,1\right], \end{array}$$ (27)

where ℏ:[0,1]×R→R is a monotonically non-decreasing function for all *g* ∈ [0, 1] and G is the Green function defined as

$$\begin{array}{c} G\left(t,g\right)=\{\begin{array}{cc} -ge^{-\tau (g-t)} & \text{if}\;\;\;0\leq g\leq t\leq 1;\\ -te^{-\tau (g-t)} & \text{if}\;\;\;0\leq t\leq g\leq 1, \end{array} \end{array}$$ (28)

τ is a constant computed in terms of R and L. Let z=C(J,R) be the space of all continuous real valued functions on J, where J = [0, 1]. Then *z* is a complete metric space with respect to the metric $J_{d}\left(\hat{a},\hat{b}\right)=\|\hat{a}-\hat{b}{\|}_{\infty }={\text{sup}}_{t\in J}\left|\hat{a}\left(t\right)-\hat{b}\left(t\right)\right|$ and so *z* is $\kappa_{JS}$-MS for κ = 1. Hereafter, we assume that (*z*, J_d_) is a $\kappa_{JS}$-MS with canonical preorder ≤ and (*z*, J_d_) is ≤-non-decreasing-complete. Define the operator ℵ: *z* → *z* as follows:

$$\begin{array}{c} ℵ\left(q\left(t\right)\right)=\int_{0}^{t}G\left(t,g\right)\hbar \left(g,q\left(g\right)\right)dg,\;t\in \left[0,1\right]. \end{array}$$ (29)

A fixed point of operator (29) is the solution of problem (26). We take into account the following hypotheses:

(H1) G: J^2^ → [0, ∞) is a continuous function;
(H2) |ℏ(t, *q*(t)) − ℏ(t, *p*(t))| ≤ |*q*(t) − *p*(t)| + 1 for all t ∈ [0, 1];
(H3) ${\left|G\right|}_{\infty }={\text{sup}}_{t\in I}\int_{0}^{t}G\left(t,g\right)\leq 1$;
(H4) ℵ is ≤-non-decreasing continuous.

**THEOREM 4.1**. *Suppose that hypothesis* (H1)-(H4) *hold. Then, the initial value problem* (26) *has a common solution in* *z*.

*Proof.* Firstly, note that for $ℑ\left(\hat{a}\right)=-\frac{1}{\hat{a}+1}$ for all $\hat{a}\in \left(0,\infty \right)$, inequality (11) is equivalent to the following for all x, y ∈ *z*:

$$\begin{array}{cc} J_{d}\left(\xi x,\xi y\right)\leq \frac{Q_{\xi }^{J_{d}}\left(x,y\right)+1}{1+\lambda Q_{\xi }^{J_{d}}\left(x,y\right)}-1 & \leq \frac{Q_{\xi }^{J_{d}}\left(x,y\right)+1}{1+\lambda Q_{\xi }^{J_{d}}\left(x,y\right)}\leq Q_{\xi }^{J_{d}}\left(x,y\right)+1. \end{array}$$ (30)

Next, for all $p,q\in O_{ℵ}(p_{0})$ and t ∈ J, we have

$$\begin{array}{cc} |ℵ(q(t))-ℵ(p(t))|= & \left|\int_{0}^{t}G\left(t,g\right)\left[\hbar \left(g,q\left(g\right)\right)-\hbar \left(g,p\left(g\right)\right)\right]dg\right|\\ \leq & \int_{0}^{t}G\left(t,g\right)|[\hbar (g,q(g))-\hbar (g,p(g))]|dg\\ \leq & \int_{0}^{t}G\left(t,g\right)\left|q\left(t\right)-p\left(t\right)+1\right|dg\\ \leq & \int_{0}^{t}G\left(t,g\right)(\text{max}\{|q\left(t\right)-p\left(t\right)|,|ℵ\left(p\left(t\right)\right)-p\left(t\right)|,|ℵ\left(q\left(t\right)\right)-q\left(t\right)|,\\ & |ℵ(q(t))-p(t)|,|ℵ(p(t))-q(t)|\}+1)dg, \end{array}$$

This implies that

$$\begin{array}{c} {\left\|ℵ\left(q\right)-ℵ\left(p\right)\right\|}_{\infty }\leq {\left|G\right|}_{\infty }(\text{max}\{\|p-q{\|}_{\infty },\|ℵ\left(p\right)-p{\|}_{\infty },\|ℵ\left(q\right)-q{\|}_{\infty },\|ℵ\left(q\right)-p{\|}_{\infty },\\ {\left\|ℵ\left(p\right)-q\right\|}_{\infty }\}+1). \end{array}$$ (31)

From (H3) and (31), we have

$$\begin{array}{c} {\left\|ℵ\left(q\right)-ℵ\left(p\right)\right\|}_{\infty }\leq {\text{max}\{\|p-q\|}_{\infty }{,\|ℵ\left(p\right)-p\|}_{\infty }{,\|ℵ\left(q\right)-q\|}_{\infty }{,\|ℵ\left(q\right)-p\|}_{\infty }{,\|ℵ\left(p\right)-q\|}_{\infty }\}+1. \end{array}$$

Hence (11) is satisfied for $ℑ\left(\hat{a}\right)=-\frac{1}{\hat{a}+1}$. Thus, all hypotheses of Theorem 2.2 are satisfied and therefore differential Eq (26) has a solution in J.

## 6 Conclusion

In this work, we establish ℑ-contractions and show some fixed point theorems for these contractive conditions in the JS-generalized metric spaces. Finally, we proved fixed point results, an existence result for the solution of the RLC circuit’s current differential equation is also established.
