# Supplementary material for: Fixed point results for ℑ-Contractions in JS-generalized metric spaces with an application
Source: PLoS One. 2025 Feb 18;20(2):e0314493. doi: 10.1371/journal.pone.0314493 (PMC11835342; doi:10.1371/journal.pone.0314493)
Supplement: Attachment — Submitted filename: Report_01.08.2024 po.pdf [file pone.0314493.s001.pdf]

A report on Fixed Point Results for Weak F-Contractions in JS Generalized Metric Spaces with an Application by Iqbal et al.

I have gone through the article; the manuscript consists of fixed-point results for weak contractions. Finally, using proven fixed point results, the existence of the solution of the RLC circuit's current differential equation is proved.

The results in this paper are intriguing, informative, and accurate. The article is sound and deserves to be published in Plos One. it will contribute positively to the literature, and the novelty of the results is high.

However, some minor changes are required as mentioned below:

1. On page 2, replace "in numerous ways. (see [4, 13, 18, 19, 20, 21, 24, 28])" with "in numerous ways (see [4, 13, 18, 19, 20, 21, 24, 28])."
2. Add a potential example of the theoretical results (Main Result) for the understanding of the readers and to show the applicability.
3. At page 2, in 3rd paragraph, add space in the following: " in[27].", "Hereafter".
4. In Remark 1.3, replace "complete As" with "complete. As"
5. There are several other typo mistakes in the article, authors are encouraged to revise the article as per suggestions.

Recommendations: **<u>MINOR REVISION</u>**
