# Supplementary material for: Fixed point results for ℑ-Contractions in JS-generalized metric spaces with an application
Source: PLoS One. 2025 Feb 18;20(2):e0314493. doi: 10.1371/journal.pone.0314493 (PMC11835342; doi:10.1371/journal.pone.0314493)
Supplement: Attachment — Submitted filename: PONE-D-24-27988.pdf [file pone.0314493.s002.pdf]

PLOS ONE

Fixed Point Results for Weak F-Contractions in JS Generalized Metric Spaces with an Application

--Manuscript Draft--

**Manuscript Number:**	PONE-D-24-27988
**Article Type:**	Research Article
**Full Title:**	Fixed Point Results for Weak F-Contractions in JS Generalized Metric Spaces with an Application
**Short Title:**	Weak F-Contractions in JS Generalized Metric Spaces
**Corresponding Author:**	Naeem Saleem<br>University of Management and Technology<br>Lahore, PAKISTAN
**Keywords:**	Generalized metric space; F-contraction; Fixed points; Weak F-contraction; Preorder; Partial order
**Abstract:**	The goal of this work is to establish weak F-contractions and to show some novel xed point theorems for these contractive conditions in the domain of JS generalized metric spaces. Finally, using proven xed point results, an existence result for the solution of the RLC circuit's current di erential equation is established.
**Order of Authors:**	Bilal Iqbal
	Naeem Saleem
	Maggie Aphane
	Asima Razzaque, PhD
**Additional Information:**	
**Question**	**Response**
**Financial Disclosure**<br><br>Enter a financial disclosure statement that describes the sources of funding for the work included in this submission. Review the submission guidelines for detailed requirements. View published research articles from *PLOS ONE* for specific examples.<br><br>This statement is required for submission and **will appear in the published article** if the submission is accepted. Please make sure it is accurate.	The author(s) received no specific funding for this work.

Powered by Editorial Manager® and ProduXion Manager® from Aries Systems Corporation

**Funded studies**<br>Enter a statement with the following details:<br>• Initials of the authors who received each award<br>• Grant numbers awarded to each author<br>• The full name of each funder<br>• URL of each funder website<br>• Did the sponsors or funders play any role in the study design, data collection and analysis, decision to publish, or preparation of the manuscript?<br><br>Did you receive funding for this work?	
**Competing Interests**<br><br>Use the instructions below to enter a competing interest statement for this submission. On behalf of all authors, disclose any competing interests that could be perceived to bias this work—acknowledging all financial support and any other relevant financial or non-financial competing interests.<br><br>This statement is required for submission and **will appear in the published article** if the submission is accepted. Please make sure it is accurate and that any funding sources listed in your Funding Information later in the submission form are also declared in your Financial Disclosure statement.<br><br>View published research articles from *PLOS ONE* for specific examples.	The authors have declared that no competing interests exist.

Powered by Editorial Manager® and ProduXion Manager® from Aries Systems Corporation

**NO authors have competing interests**<br><br>Enter: *The authors have declared that no competing interests exist.*<br><br>**Authors with competing interests**<br><br>Enter competing interest details beginning with this statement:<br><br>*I have read the journal's policy and the authors of this manuscript have the following competing interests: [insert competing interests here]*<br><br>* typeset	
**Ethics Statement**<br><br>Enter an ethics statement for this submission. This statement is required if the study involved:<br><br>• Human participants<br>• Human specimens or tissue<br>• Vertebrate animals or cephalopods<br>• Vertebrate embryos or tissues<br>• Field research<br><br>Write "N/A" if the submission does not require an ethics statement.<br><br>General guidance is provided below. Consult the submission guidelines for detailed instructions. **Make sure that all information entered here is included in the Methods section of the manuscript.**	NA

**Format for specific study types**<br><br>**Human Subject Research (involving human participants and/or tissue)**<br>• Give the name of the institutional review board or ethics committee that approved the study<br>• Include the approval number and/or a statement indicating approval of this research<br>• Indicate the form of consent obtained (written/oral) or the reason that consent was not obtained (e.g. the data were analyzed anonymously)<br><br>**Animal Research (involving vertebrate animals, embryos or tissues)**<br>• Provide the name of the Institutional Animal Care and Use Committee (IACUC) or other relevant ethics board that reviewed the study protocol, and indicate whether they approved this research or granted a formal waiver of ethical approval<br>• Include an approval number if one was obtained<br>• If the study involved *non-human primates*, add *additional details* about animal welfare and steps taken to ameliorate suffering<br>• If anesthesia, euthanasia, or any kind of animal sacrifice is part of the study, include briefly which substances and/or methods were applied<br><br>**Field Research**<br><br>Include the following details if this study involves the collection of plant, animal, or other materials from a natural setting:<br>• Field permit number<br>• Name of the institution or relevant body that granted permission	
**Data Availability**<br><br>Authors are required to make all data underlying the findings described fully available, without restriction, and from the time of publication. PLOS allows rare exceptions to address legal and ethical concerns. See the PLOS Data Policy and FAQ for detailed information.	No - some restrictions will apply

A Data Availability Statement describing where the data can be found is required at submission. Your answers to this question constitute the Data Availability Statement and **will be published in the article**, if accepted.<br><br>**Important:** Stating 'data available on request from the author' is not sufficient. If your data are only available upon request, select 'No' for the first question and explain your exceptional situation in the text box.<br><br>Do the authors confirm that all data underlying the findings described in their manuscript are fully available without restriction?	
**Describe where the data may be found in full sentences. If you are copying our sample text, replace any instances of XXX with the appropriate details.**<br><br>• If the data are **held or will be held in a public repository**, include URLs, accession numbers or DOIs. If this information will only be available after acceptance, indicate this by ticking the box below. For example: *All XXX files are available from the XXX database (accession number(s) XXX, XXX.).*<br>• If the data are all contained **within the manuscript and/or Supporting Information files**, enter the following: *All relevant data are within the manuscript and its Supporting Information files.*<br>• If neither of these applies but you are able to provide **details of access elsewhere**, with or without limitations, please do so. For example:<br><br>*Data cannot be shared publicly because of [XXX]. Data are available from the XXX Institutional Data Access / Ethics Committee (contact via XXX) for researchers who meet the criteria for access to confidential data.*<br><br>*The data underlying the results presented in the study are available from (include the name of the third party*	No data was used during this study.

and contact information or URL).<br>• This text is appropriate if the data are owned by a third party and authors do not have permission to share the data.<br><br>* typeset	
Additional data availability information:	Tick here if your circumstances are not covered by the questions above and you need the journal’s help to make your data available.; Tick here if the URLs/accession numbers/DOIs will be available only after acceptance of the manuscript for publication so that we can ensure their inclusion before publication.

Powered by Editorial Manager® and ProduXion Manager® from Aries Systems Corporation

Manuscript Click here to access/download;Manuscript;Paper-III Revise.pdf

Fixed Point Results for Weak $\Im$-Contractions in JS Generalized Metric Spaces with an Application

Bilal Iqbal[1], *Naeem Saleem*[1,2*], *Maggie Aphane*[2], *Asima Razzaque* [3,4]

[1] *Department of Mathematics, University of Management and Technology, Lahore, Pakistan*

E-mail address: naeem.saleem2@gmail.com (N.S.), bimirza91@gmail.com (B.I.)

[2] *Department of Mathematics and Applied Mathematics, Sefako Makgatho Health Sciences University, Ga-Rankuwa, Pretoria, Medunsa-0204, South Africa*

E-mail address: maggie.aphane@smu.ac.za (M.A.)

[3] *Department of Basic Sciences, Preparatory Year, King Faisal University, Al-Ahsa 31982, Saudi Arabia*

[4] *Department of Mathematics, College of Science, King Faisal University, Al-Ahsa 31982, Saudi Arabia*

E-mail address: arazzaque@kfu.edu.sa

Correspondence: naeem.saleem2@gmail.com (N.S.); arazzaque@kfu.edu.sa (A.R.)

Abstract

The goal of this work is to establish weak $\Im$-contractions and to show some novel fixed point theorems for these contractive conditions in the domain of JS generalized metric spaces. Finally, using proven fixed point results, an existence result for the solution of the RLC circuit's current differential equation is established.

MSC: 54H25; 47H10; 46T99.

Keywords: Generalized metric space; $\Im$-contraction; Fixed points; Weak $\Im$-contraction; Preorder; Partial order.

1. Introduction

The Banach fixed point theorem [3] served as the inspiration for metric fixed point theory. Because this approach has many applications in several disciplines, several authors have expanded it in many ways. Wardowski [30] provides one such astonishing and significant generalization. He introduced the notion of $\Im$-contraction as follows:

Definition 1.1. Let $(\mathcal{Z}, m)$ be a metric space. A mapping $\xi : \mathcal{Z} \to \mathcal{Z}$ is said to be an $\Im$-contraction, if there is $\Im \in \Delta(\Im)$ and $\lambda > 0$ such that for all $\varpi, \varsigma \in \mathcal{Z}$

$$\lambda + \Im(m(\xi\varpi, \xi\varsigma)) \leq \Im(m(\varpi, \varsigma)), \tag{1.1}$$

where $\Delta(\Im)$ is the family of all mappings $\Im : (0, +\infty) \to \mathbb{R}$ obeying the following conditions:

($\Im_1$) $\Im(\varpi) < \Im(\varsigma)$ for all $\varpi < \varsigma$;
($\Im_2$) for all sequences $\{\eta_p\} \subseteq (0, +\infty)$, $\lim_{p\to\infty} \eta_p = 0$, if and only if $\lim_{p\to\infty} \Im(\eta_p) = -\infty$;
($\Im_3$) there exists $0 < \ell < 1$ such that $\lim_{\eta\to 0^+} \eta^\ell \Im(\eta) = 0$.

Wardowski's result is given as follows:

THEOREM 1.1 ([30]). *Let* $(\mathcal{Z}, \mathfrak{m})$ *be a complete metric space and* $\xi : \mathcal{Z} \to \mathcal{Z}$ *be* $\Im$-*contraction. Then* $\varpi^* \in \mathcal{Z}$ *is a unique fixed point of* ξ *and for each* $\varpi_0 \in \mathcal{Z}$ *a sequence* $\{\xi^p \varpi_0\}_{p\in\mathbb{N}}$ *is convergent to* ϖ^*.

In [26], Secelean demonstrated that condition $(\Im_2)$ may be overtaken by an equivalent and simpler one.

$(\Im_2')$ $\lim_{t\to 0^+} \Im(t) = -\infty$.

Lemma 1.1. *Let* $\Im : (0, \infty) \to \mathbb{R}$ *be a function obeying* $(\Im_1)$ *&* $(\Im_2')$*, then for all sequence* $\{t_p\} \subseteq (0, 1)$

$$\Im(t_p) \to -\infty \quad \textit{implies} \quad t_p \to 0.$$

Following that, Piri and Kumam [23] established Wardowski's theorem utilising $(\Im_2')$ and the continuity rather than $(\Im_2)$ and $(\Im_3)$, respectively. Wardowski [31] later proved a fixed point theorem for $\Im$-contractions when λ is treated as a function. Recently, other authors demonstrated (in various methods) Wardowski's original results in the absence of both criterias $(\Im_2)$ and $(\Im_3)$ (see, [17, 22]). To more in this direction, consult [1, 7, 8, 9, 10, 11, 29, 32]. Very recently, Derouiche and Ramoul [6] used a relaxed version of $(\Im_2)$ and also dropped $(\Im_3)$ to establish some new fixed point results in the context of b-metric spaces.
On the other side, the concept of standard metric space is generalized in numerous ways. (see [4, 13, 18, 19, 20, 21, 24, 28]). Jleli and Samet [14] recapitulated a huge class of topological spaces by introducing the most prevailing generalizations of metric spaces. More far, in [15], Karapinar *et al.* achieved fixed point theorems under very general contractive conditions and Altun *et al.* [2] proved a Feng-Liu's type fixed point theorem in JS generalized metric spaces. While, in [5], Dumitrescu and Pitea presented extensions of some classic results regarding the existence and uniqueness of fixed points of operators fulfilling generalized contractive conditions in the setting of JS generalized metric spaces. Quite recently, Saleem *et al.* [25] proved some new fixed point theorems, coincidence point theorems and common fixed point theorems for multivalued $\Im$-contractions in the framework of JS generalized metric spaces. Afterwards, Iqbal *et al.* [12] derived the coincidence fixed point and common fixed point results for $\Im$-type mappings with regard to JS generalized metric spaces.
A binary relation on $\mathcal{Z}$ is a non-empty subset $\sim_{\mathcal{R}}$ of the Cartesian product $\mathcal{Z} \times \mathcal{Z}$. For ease of use, we designate $\varpi \sim_{\mathcal{R}} \varsigma$ if $(\varpi, \varsigma) \in \sim_{\mathcal{R}}$. The concepts of antisymmetry, preorder, reflexivity, transitivity, and partial order can be found in[27]. The trivial preorder on $\mathcal{Z}$ is denoted by $\sim_{\mathcal{R}_\mathcal{Z}}$, and is given by $\varpi \sim_{\mathcal{R}_\mathcal{Z}} \varsigma$ for each $\varpi, \varsigma \in \mathcal{Z}$. Hereafter, $\mathbb{R}$ and $\mathbb{N}$ demonstrate the set the set of real numbers and the set of non-negative integers, respectively. Let $\mathcal{Z}$ be a non-empty set and $\mathcal{J}_d : \mathcal{Z} \times \mathcal{Z} \to [0, \infty]$ be a given mapping. Following Jleli and Samet

[14], for every $\varpi \in \mathcal{Z}$, define the set

$$\mathcal{C}(\mathcal{J}_d, \mathcal{Z}, \varpi) = \left\{ \{\varpi_p\} \subset \mathcal{Z} : \lim_{p\to\infty} \mathcal{J}_d(\varpi_p, \varpi) = 0 \right\}. \tag{1.2}$$

Definition 1.2 ([14]). Let $\mathcal{Z}$ be a non-empty set and $\mathcal{J}_d : \mathcal{Z} \times \mathcal{Z} \to [0, \infty]$ be a function that complies with the following criteria for all $\varpi, \varsigma \in \mathcal{Z}$:

($\mathcal{J}_{d1}$) $\mathcal{J}_d(\varpi, \varsigma) = 0$ implies $\varpi = \varsigma$;
($\mathcal{J}_{d2}$) $\mathcal{J}_d(\varpi, \varsigma) = \mathcal{J}_d(\varsigma, \varpi)$;
($\mathcal{J}_{d3}$) there exist $\kappa > 0$ such that $(\varpi, \varsigma) \in \mathcal{Z} \times \mathcal{Z}$, $\{\varpi_p\} \in \mathcal{C}(\mathcal{J}_d, \mathcal{Z}, \varpi)$ implies

$$\mathcal{J}_d(\varpi, \varsigma) \leq \kappa \lim_{p\to\infty} \sup \mathcal{J}_d(\varpi_p, \varsigma). \tag{1.3}$$

Then $\mathcal{J}_d$ is called a JS generalized metric and the pair $(\mathcal{Z}, \mathcal{J}_d)$ is called a JS generalized metric space. We renamed it as κ-JS generalized metric space (in short, a $\kappa_{\mathcal{JS}}$-MS).

Remark 1.1 ([14]). If the set $\mathcal{C}(\mathcal{J}_d, \mathcal{Z}, \varpi)$ is empty for every $\varpi \in \mathcal{Z}$, then $(\mathcal{Z}, \mathcal{J}_d)$ is a $\kappa_{\mathcal{JS}}$-MS if and only if ($\mathcal{J}_{d1}$) and ($\mathcal{J}_{d2}$) are satisfied.

Many examples of $\kappa_{\mathcal{JS}}$-MS can be found in [14], [15] and [25].

Example 1.1 ([14]). (1) *A modular metric space* $(\mathcal{Z}, \rho)$ *is a* $\rho_{\mathcal{JS}}$*-MS.*
(2) *A standard metric space is a* $1_{\mathcal{JS}}$*-MS.*
(3) *A 2-metric space is a* $2_{\mathcal{JS}}$*-MS.*

Definition 1.3. [14] Let $(\mathcal{Z}, \mathcal{J}_d)$ be a $\kappa_{\mathcal{JS}}$-MS and $\varpi \in \mathcal{Z}$.

(1) A sequence $\{\varpi_p\} \subseteq \mathcal{Z}$ is said to be $\mathcal{J}_d$-convergent and $\mathcal{J}_d$-converges to ϖ if $\{\varpi_p\} \in \mathcal{C}(\mathcal{J}_d, \mathcal{Z}, \varpi)$. In such case, we will write $\{\varpi_p\} \xrightarrow{\mathcal{J}_d} \varpi$.
(2) a sequence $\{\varpi_p\} \subseteq \mathcal{Z}$ is said to be $\mathcal{J}_d$-Cauchy if

$$\lim_{p,q\to\infty} \mathcal{J}_d(\varpi_p, \varpi_{p+q}) = 0. \tag{1.4}$$

(3) A $\kappa_{\mathcal{JS}}$-MS $(\mathcal{Z}, \mathcal{J}_d)$ is said to be complete if every $\mathcal{J}_d$-Cauchy sequence in $\mathcal{Z}$ is $\mathcal{J}_d$-convergent.

Proposition 1.1 ([14]). *Let* $(\mathcal{Z}, \mathcal{J}_d)$ *be a* $\kappa_{\mathcal{JS}}$*-MS,* $\{\varpi_p\}$ *be a sequence in* $\mathcal{Z}$ *and* $(\varpi, \varsigma) \in \mathcal{Z} \times \mathcal{Z}$*. If* $\{\varpi_p\}$ *is* $\mathcal{J}_d$*-convergent and* $\mathcal{J}_d$*-converges to* ϖ *and* $\{\varpi_p\}$ $\mathcal{J}_d$*-converges to* ς*, then* $\varpi = \varsigma$*.*

Remark 1.2. $\mathcal{J}_d$-Cauchy sequence is defined Jleli and Samet as

$$\lim_{p,q\to\infty} \mathcal{J}_d(\varpi_p, \varpi_q) = 0. \tag{1.5}$$

Clearly, (1.5) implies (1.4), the opposite, however, need not be true [15]. From here on, we assume that $\mathcal{J}_d$-Cauchy sequences are given by (1.5).

Definition 1.4 ([15]). Let $(\mathcal{Z}, \mathcal{J}_d)$ be a $\kappa_{\mathcal{JS}}$-MS and $\xi : \mathcal{Z} \to \mathcal{Z}$. For $\varpi_0 \in \mathcal{Z}$, denote $\delta(\mathcal{J}_d, \xi, \varpi_0)$, the $\mathcal{J}_d$-diameter of the orbit of ϖ_0 by ξ, $\mathcal{O}_\xi(\varpi_0) = \{\xi^p \varpi_0 : p \in \mathbb{N}\}$, and is defined as,

$$\delta(\mathcal{J}_d, \xi, \varpi_0) = \sup(\{\mathcal{J}_d(\xi^p \varpi_0, \xi^q \varpi_0) : q, p \in \mathbb{N}\}). \tag{1.6}$$

Definition 1.5 ([15])**.** Let $\sim_{\mathcal{R}}$ be a binary relation on $\kappa_{\mathcal{JS}}$-MS $(\mathcal{Z}, \mathcal{J}_d)$. A sequence $\{\varpi_p\} \subseteq \mathcal{Z}$ is $\sim_{\mathcal{R}}$-non-decreasing if $\varpi_p \sim_{\mathcal{R}} \varpi_{p+1}$ for each $p \in \mathbb{N}$.

Definition 1.6 ([15])**.** A $\kappa_{\mathcal{JS}}$-MS $(\mathcal{Z}, \mathcal{J}_d)$ is called $\sim_{\mathcal{R}}$-non-decreasing complete if every $\sim_{\mathcal{R}}$-non-decreasing and $\mathcal{J}_d$-Cauchy sequence is $\mathcal{J}_d$-convergent in $\mathcal{Z}$.

Remark 1.3. Notice that every complete $\kappa_{\mathcal{JS}}$-MS is also $\sim_{\mathcal{R}}$-non-decreasing complete As evidenced by the case below, the contrary is untrue.

Example 1.2. *Let $\mathcal{Z} = (0, 1]$ furnished with the Euclidean metric $m(\varpi, \varsigma) = |\varpi - \varsigma|$ for each $\varpi, \varsigma \in \mathcal{Z}$. Define a binary relation $\sim_{\mathcal{R}}$ on $\mathcal{Z}$ by*

$$\varpi \sim_{\mathcal{R}} \varsigma \text{ if } 0 < \varpi \leq \varsigma \leq 1.$$

Then $(\mathcal{Z}, m)$ is $\sim_{\mathcal{R}}$-non-decreasing complete, however, the metric space is not complete.

Definition 1.7 ([15])**.** Let $(\mathcal{Z}, \mathcal{J}_d)$ be a $\kappa_{\mathcal{JS}}$-MS. A mapping $\xi : \mathcal{Z} \to \mathcal{Z}$ is $\sim_{\mathcal{R}}$-non-decreasing-continuous at $\nu \in \mathcal{Z}$ if $\{\xi\varpi_p\} \in \mathcal{C}(\mathcal{J}_d, \mathcal{Z}, \psi\nu)$ for each $\sim_{\mathcal{R}}$-non-decreasing sequence $\{\varpi_p\} \in \mathcal{C}(\mathcal{J}_d, \mathcal{Z}, \nu)$. A mapping ξ is $\sim_{\mathcal{R}}$-non-decreasing-continuous if it is $\sim_{\mathcal{R}}$-non-decreasing-continuous at every point of $\mathcal{Z}$.

Remark 1.4. [15] Every continuous mapping is also $\sim_{\mathcal{R}}$-non-decreasing-continuous, while the reverse is generally false, as seen in Example 4.6 of [15].

By getting inspiration from the work of Karapinar *et al.* [15], here, we prove fixed point theorems for weak $\Im$-contractions in the context of JS generalized metric space.

2. Fundamental Results

Let $(\mathcal{Z}, \mathcal{J}_d)$ be a $\kappa_{\mathcal{JS}}$-MS and let ξ be a self-mapping on $\mathcal{Z}$. Throughout this section, we denote, for all $\varpi, \varsigma \in \mathcal{Z}$,

$$\mathcal{Q}_{\xi}^{\mathcal{J}_d}(\varpi, \varsigma) = \max\{\mathcal{J}_d(\varpi, \varsigma), \mathcal{J}_d(\varpi, \xi\varpi), \mathcal{J}_d(\varsigma, \xi\varsigma), \mathcal{J}_d(\varpi, \xi\varsigma), \mathcal{J}_d(\varsigma, \xi\varpi)\}.$$

Following [15], define for given $p_0 \in \mathbb{N}$,

$$\delta_{p_0}(\mathcal{J}_d, \xi, \varpi_0) = \sup(\{\mathcal{J}_d(\xi^p\varpi_0, \xi^q\varpi_0) : q, p \in \mathbb{N}, q, p \geq p_0\}). \tag{2.1}$$

and

$$\delta(\mathcal{J}_d, \xi, \varpi_0) = \delta_0(\mathcal{J}_d, \xi, \varpi_0) = \sup(\{\mathcal{J}_d(\xi^p\varpi_0, \xi^q\varpi_0) : q, p \in \mathbb{N}\}). \tag{2.2}$$

By the symmetry of $\mathcal{J}_d$, we can alternatively express

$$\delta_{p_0}(\mathcal{J}_d, \xi, \varpi_0) = \sup(\{\mathcal{J}_d(\xi^p\varpi_0, \xi^q\varpi_0) : q, p \in \mathbb{N}, q \geq p \geq p_0\}). \tag{2.3}$$

Notice that if $q, p \in \mathbb{N}$ satisfy $q \geq p$, then

$$\delta_q(\mathcal{J}_d, \xi, \varpi_0) \leq \delta_p(\mathcal{J}_d, \xi, \varpi_0) \leq \delta(\mathcal{J}_d, \xi, \varpi_0). \tag{2.4}$$

Lemma 2.1. *Let $(\mathcal{Z}, \mathcal{J}_d)$ be a $\kappa_{\mathcal{JS}}$-MS with a preorder $\sim_{\mathcal{R}}$ and let $\xi : \mathcal{Z} \to \mathcal{Z}$ be an $\sim_{\mathcal{R}}$-non-decreasing mapping. Let $\varpi_0 \in \mathcal{Z}$ be a point such that ϖ_0 and $\xi\varpi_0$ are $\sim_{\mathcal{R}}$-comparable. Assume, there is a function $\Im : (0, \infty) \to \mathbb{R}$ such that*

$$\xi\varpi \neq \xi\varsigma \text{ implies } \lambda + \Im(\mathcal{J}_d(\xi\varpi, \xi\varsigma)) \leq \Im(\mathcal{Q}_\xi^{\mathcal{J}_d}(\varpi, \varsigma)), \tag{2.5}$$

for $\varpi, \varsigma \in \mathcal{Z}$ satisfying $\varpi \sim_{\mathcal{R}} \varsigma$ and $\lambda > 0$. Then (2.5) holds for each $\varpi, \varsigma \in \mathcal{O}_\xi(\varpi_0)$.

Proof. Consider the Picard sequence $\{\varpi_p = \xi\varpi_{p-1} = \xi^p\varpi_0\}_{p\in\mathbb{N}}$ of ξ based on ϖ_0. Suppose that $\varpi_0 \sim_{\mathcal{R}} \varpi_1$, as ξ is $\sim_{\mathcal{R}}$-non-decreasing, then $\varpi_1 = \xi\varpi_0 \sim_{\mathcal{R}} \xi\varpi_1 = \varpi_2$. Repeating this argument, we get, $\varpi_p \sim_{\mathcal{R}} \varpi_{p+1}$ for every $p \in \mathbb{N}$. Since $\sim_{\mathcal{R}}$ is a preorder, then $\varpi_p \sim_{\mathcal{R}} \varpi_q$ for all $q, p \in \mathbb{N}$ such that $p \leq q$. Furthermore, as condition (2.5) is symmetric on ϖ and ς, then (2.5) holds for each ϖ_p and ϖ_q (being $q, p \in \mathbb{N}$ arbitrary), so it holds for each $\varpi, \varsigma \in \mathcal{O}_\xi(\varpi_0)$. □

Lemma 2.2. *Let $(\mathcal{Z}, \mathcal{J}_d)$ be a $\kappa_{\mathcal{JS}}$-MS and let $\xi : \mathcal{Z} \to \mathcal{Z}$ be a mapping. Let $\varpi_0 \in \mathcal{Z}$ be a point for which there exists $p_0 \in \mathbb{N}$ such that $\delta p_0(\mathcal{J}_d, \xi, \varpi) < \infty$. Assume, there is a non-decreasing function $\Im : (0, \infty) \to \mathbb{R}$ obeying*

$(\Im_{\mathcal{J}_d})$: $\Im(\sup M) = \sup \Im(M)$ *for all $M \subset (0, \infty)$ with $\sup M > 0$*

and

$$\xi\varpi \neq \xi\varsigma \text{ implies } \lambda + \Im(\mathcal{J}_d(\xi\varpi, \xi\varsigma)) \leq \Im(\mathcal{Q}_\xi^{\mathcal{J}_d}(\varpi, \varsigma)), \tag{2.6}$$

for all $\varpi, \varsigma \in \mathcal{O}_\xi(\varpi_0)$ and $\lambda > 0$. Then

$$\delta_{\ell+1}(\mathcal{J}_d, \xi, \varpi_0) \leq \delta_\ell(\mathcal{J}_d, \xi, \varpi_0) - \lambda, \quad \text{for all} \quad \ell \in \mathbb{N}, \ell \geq p_0.$$

In particular,

$$\delta_{p_0+\ell}(\mathcal{J}_d, \xi, \varpi_0) \leq \delta_{p_0}(\mathcal{J}_d, \xi, \varpi_0) - \ell\lambda, \quad \text{for all} \quad \ell \in \mathbb{N}, \ell \geq p_0.$$

Proof. Let $\ell \in \mathbb{N}$ be such that $\ell \geq p_0$. From (2.4), we have

$$\delta_{\ell+1}(\mathcal{J}_d, \xi, \varpi_0) \leq \delta_\ell(\mathcal{J}_d, \xi, \varpi_0) \leq \delta_{p_0}(\mathcal{J}_d, \xi, \varpi_0) < \infty.$$

Let $q, p \in \mathbb{N}$ be such that $p \geq q \geq \ell + 1$. Denote

$$\Omega_\ell = \{\mathcal{J}_d(\xi^r\varpi_0, \xi^s\varpi_0) : r, s \in \mathbb{N},\ r, s \geq \ell\},$$

then

$$\mathcal{J}_d(\xi^{p-1}\varpi_0, \xi^{q-1}\varpi_0), \mathcal{J}_d(\xi^{p-1}\varpi_0, \xi^p\varpi_0), \mathcal{J}_d(\xi^{q-1}\varpi_0, \xi^q\varpi_0), \mathcal{J}_d(\xi^{p-1}\varpi_0, \xi^q\varpi_0), \mathcal{J}_d(\xi^{q-1}\varpi_0, \xi^p\varpi_0) \in \Omega_\ell.$$

Hence

$$\begin{aligned} &\max\{\mathcal{J}_d(\xi^{p-1}\varpi_0, \xi^{q-1}\varpi_0), \mathcal{J}_d(\xi^{p-1}\varpi_0, \xi^p\varpi_0), \mathcal{J}_d(\xi^{q-1}\varpi_0, \xi^q\varpi_0), \mathcal{J}_d(\xi^{p-1}\varpi_0, \xi^q\varpi_0), \mathcal{J}_d(\xi^{q-1}\varpi_0, \xi^p\varpi_0)\} \\ \leq\ &\sup \Omega_\ell \\ =\ &\delta_\ell(\mathcal{J}_d, \xi, \varpi_0). \end{aligned} \tag{2.7}$$

From (2.7) and (2.7), we obtain

$$\begin{aligned} \lambda + \Im(\mathcal{J}_d(\xi^p\varpi_0, \xi^q\varpi_0)) &\leq \Im(\mathcal{Q}_\xi^{\mathcal{J}_d}(\xi^{p-1}\varpi_0, \xi^{q-1}\varpi_0)) \\ &\leq \Im(\delta_\ell(\mathcal{J}_d, \xi, \varpi_0)). \end{aligned} \tag{2.8}$$

By the virtue of $(\Im_{\jmath_d})$ and (2.8), we get

$$\begin{aligned}\Im(\delta_{\ell+1}(\jmath_d,\xi,\varpi_0)) &= \Im(\sup\{\jmath_d(\xi^p\varpi_0,\xi^q\varpi_0) : q,p\in\mathbb{N},\ \ell+1\le n\le p\})\\ &= \sup\{\Im(\jmath_d(\xi^p\varpi_0,\xi^q\varpi_0)) : q,p\in\mathbb{N},\ \ell+1\le n\le p\}\\ &\le \sup\{\Im(\delta_\ell(\jmath_d,\xi,\varpi_0))-\lambda : q,p\in\mathbb{N},\ \ell+1\le n\le p\}\\ &\le \Im(\delta_\ell(\jmath_d,\xi,\varpi_0))-\lambda.\end{aligned}\tag{2.9}$$

Continuing this argument and recognizing that $\Im$ is non-decreasing, we get for all $\ell\in\mathbb{N}$

$$\begin{aligned}\Im(\delta_{p_0+\ell}(\jmath_d,\xi,\varpi_0)) &\le \Im(\delta_{p_0+\ell-1}(\jmath_d,\xi,\varpi_0))-\lambda\\ &\le \Im(\delta_{p_0+\ell-2}(\jmath_d,\xi,\varpi_0))-2\lambda\\ &\ \vdots\\ &\le \Im(\delta_{p_0}(\jmath_d,\xi,\varpi_0))-\ell\lambda.\end{aligned}$$

□

3. Main Results

THEOREM 3.1. *Let $(\mathcal{Z},\jmath_d)$ be a $\kappa_{\jmath\mathcal{S}}$-MS with a preorder $\sim_{\mathcal{R}}$ and let $\xi:\mathcal{Z}\to\mathcal{Z}$ be an $\sim_{\mathcal{R}}$-non-decreasing mapping. Let $\varpi_0\in\mathcal{Z}$ be a point such that $\varpi_0\sim_{\mathcal{R}}\xi\varpi_0$ and $\delta p_0(\jmath_d,\xi,\varpi)<\infty$ for some $p_0\in\mathbb{N}$. Assume, there is a non-decreasing function $\Im:(0,\infty)\to\mathbb{R}$ obeying $(\Im_2')$, $(\Im_{\jmath_d})$ and (2.5) for all $\varpi,\varsigma\in\mathcal{O}_\xi(\varpi_0)$ and $\lambda>0$. Then the sequence $\{\xi^p\varpi_0\}_{p\in\mathbb{N}}$ based on ϖ_0 is $\sim_{\mathcal{R}}$-non-decreasing and $\jmath_d$-Cauchy sequence.*
Furthermore, if $(\mathcal{Z},\jmath_d)$ is $\sim_{\mathcal{R}}$-non-decreasing-complete, then $\{\xi^p\varpi_0\}_{p\in\mathbb{N}}$ $\jmath_d$-converges to a point $\nu\in\mathcal{Z}$ that obeys

$$\jmath_d(\nu,\nu)=0.$$

Additionally, if ξ is $\sim_{\mathcal{R}}$-non-decreasing-continuous, then $\nu\in\xi\nu$.

Proof. Consider the Picard sequence $\{\varpi_p=\xi\varpi_{p-1}=\xi^p\varpi_0\}_{p\in\mathbb{N}}$ of ξ based on ϖ_0. As shown in the proof of Lemma 2.1, $\{\xi^p\varpi_0\}_{p\in\mathbb{N}}$ is $\sim_{\mathcal{R}}$-non-decreasing. If $\xi^p\varpi_0=\xi^q\varpi_0$, then $\jmath_d(\varpi_p,\varpi_q)=0$ for every $q,p\ge p_0$. In particular,

$$\lim_{p,q\to\infty}\jmath_d(\varpi_p,\varpi_q)=0.$$

Consider $\xi^p\varpi_0\neq\xi^q\varpi_0$ and $q,p\in\mathbb{N},p_0\le p<q$ for some $p_0\in\mathbb{N}$, then by using Lemma 2.2, we have

$$\Im(\jmath_d(\xi^p\varpi_0,\xi^q\varpi_0))\le\Im(\delta_{q+p}(\jmath_d,\xi,\varpi_0))\le\Im(\delta_q(\jmath_d,\xi,\varpi_0))-p\lambda.\tag{3.1}$$

Letting limit in (3.1) as $p,q\to\infty$, we have

$$\lim_{p,q\to\infty}\Im(\jmath_d(\xi^p\varpi_0,\xi^q\varpi_0))=\infty.\tag{3.2}$$

Taking into account of $(\Im_2')$, we have

$$\lim_{p,q\to\infty}\jmath_d(\xi^p\varpi_0,\xi^q\varpi_0)=0.$$

Hence $\{\xi^p \varpi_0\}_{p\in\mathbb{N}}$ $\mathcal{J}_d$-Cauchy sequence. Since $(\mathcal{Z}, \mathcal{J}_d)$ is $\sim_{\mathcal{R}}$-non-decreasing-complete, there exists $\nu \in \mathcal{Z}$ such that $\{\varpi_p\} \xrightarrow{\mathcal{J}_d} \nu$. By using $(\mathcal{J}_{d3})$, we get

$$0 \leq \mathcal{J}_d(\nu, \nu) \leq \kappa \lim_{p\to\infty} \sup \mathcal{J}_d(\varpi_p, \nu) = 0, \tag{3.3}$$

which implies $\mathcal{J}_d(\nu, \nu) = 0$.
Moreover, as we additionally assume that ξ is $\sim_{\mathcal{R}}$-non-decreasing-continuous, then

$$\{\varpi_{p+1} = \xi^p \varpi_0 = \xi \varpi_p\} \xrightarrow{\mathcal{J}_d} \xi\nu.$$

Proposition 1.1 guarantees that $\xi\nu = \nu$, so ν is a fixed point of ξ. □

In next theorem, we replace the condition of $(\Im_2')$ by the continuity of $\Im$ in Theorem 3.1.

THEOREM 3.2. *Let $(\mathcal{Z}, \mathcal{J}_d)$ be a $\kappa_{\mathcal{J}_S}$-MS with a preorder $\sim_{\mathcal{R}}$ and let $\xi : \mathcal{Z} \to \mathcal{Z}$ be an $\sim_{\mathcal{R}}$-non-decreasing self-mapping. Let $\varpi_0 \in \mathcal{Z}$ be a point such that $\varpi_0 \sim_{\mathcal{R}} \xi\varpi_0$ and $\delta p_0(\mathcal{J}_d, \xi, \varpi) < \infty$ for some $p_0 \in \mathbb{N}$. Assume, there exists a continuous non-decreasing function $\Im : (0, \infty) \to \mathbb{R}$ satisfying (2.5) for all $\varpi, \varsigma \in \mathcal{O}_\xi(\varpi_0)$, $\lambda > 0$ and the following:*

$$\lim_{p_0+\ell\to\infty} \delta_{p_0+\ell}(\mathcal{J}_d, \xi, \varpi_0) = \lim_{p_0\to\infty} \delta_{p_0}(\mathcal{J}_d, \xi, \varpi_0) \quad \text{for arbitrary } \ell \in \mathbb{N} \text{ such that } \ell \geq p_0.$$

Then the sequence $\{\xi^p \varpi_0\}_{p\in\mathbb{N}}$ based on ϖ_0 is $\sim_{\mathcal{R}}$-non-decreasing and $\mathcal{J}_d$-Cauchy sequence. Furthermore, if $(\mathcal{Z}, \mathcal{J}_d)$ is $\sim_{\mathcal{R}}$-non-decreasing-complete, then $\{\xi^p \varpi_0\}_{p\in\mathbb{N}}$ $\mathcal{J}_d$-converges to a point $\nu \in \mathcal{Z}$ that satisfies

$$\mathcal{J}_d(\nu, \nu) = 0.$$

Additionally, if ξ is $\sim_{\mathcal{R}}$-non-decreasing-continuous, then $\nu \in \xi\nu$.

Proof. Consider the Picard sequence $\{\varpi_p = \xi\varpi_{p-1} = \xi^p \varpi_0\}_{p\in\mathbb{N}}$ of ξ based on ϖ_0. As shown in the proof of Lemma 2.1, $\{\xi^p \varpi_0\}_{p\in\mathbb{N}}$ is $\sim_{\mathcal{R}}$-non-decreasing. If $\delta_{p_0}(\mathcal{J}_d, \xi, \varpi_0) = 0$, then $\mathcal{J}_d(\varpi_p, \varpi_q) = 0$ for all $q, p \geq p_0$. In particular,

$$\lim_{q,p\to\infty} \mathcal{J}_d(\varpi_p, \varpi_q) = 0.$$

Consider $\delta_{p_0}(\mathcal{J}_d, \xi, \varpi_0) > 0$ and $q, p \in \mathbb{N}, p_0 \leq p < q$ for some $p_0 \in \mathbb{N}$. Denote

$$\Omega_{p_0} = \{\mathcal{J}_d(\xi^r \varpi_0, \xi^s \varpi_0) : r, s \in \mathbb{N},\ r, s \geq p_0\},$$

then

$$\mathcal{J}_d(\xi^{p-1}\varpi_0, \xi^{q-1}\varpi_0), \mathcal{J}_d(\xi^{p-1}\varpi_0, \xi^p\varpi_0), \mathcal{J}_d(\xi^{q-1}\varpi_0, \xi^q\varpi_0), \mathcal{J}_d(\xi^{p-1}\varpi_0, \xi^q\varpi_0), \mathcal{J}_d(\xi^{q-1}\varpi_0, \xi^p\varpi_0) \in \Omega_{p_0}.$$

Hence

$$\begin{aligned} &\max\{\mathcal{J}_d(\xi^{p-1}\varpi_0, \xi^{q-1}\varpi_0), \mathcal{J}_d(\xi^{p-1}\varpi_0, \xi^p\varpi_0), \mathcal{J}_d(\xi^{q-1}\varpi_0, \xi^q\varpi_0), \mathcal{J}_d(\xi^{p-1}\varpi_0, \xi^q\varpi_0), \mathcal{J}_d(\xi^{q-1}\varpi_0, \xi^p\varpi_0)\} \\ &\leq \sup \Omega_{p_0} \\ &= \delta_{p_0}(\mathcal{J}_d, \xi, \varpi_0). \end{aligned} \tag{3.4}$$

Assume that $\lim_{p_0+\ell\to\infty}\delta_{p_0+\ell}(\mathcal{J}_d,\xi,\varpi_0)=\lim_{p_0\to\infty}\delta_{p_0}(\mathcal{J}_d,\xi,\varpi_0)=\mho$ for $\ell\in\mathbb{N}$ be an arbitrary such that $\ell\geq p_0$. Then, by using inequalities (2.5) and (3.4) and continuity of $\Im$, we obtain

$$\begin{aligned}\lambda+\Im(\mho)&=\lambda+\Im\left(\lim_{p_0+\ell\to\infty}\delta_{p_0+\ell}(\mathcal{J}_d,\xi,\varpi_0)\right)\\&=\lambda+\Im\left(\limsup_{p,q\to\infty}\mathcal{J}_d(\xi^p\varpi_0,\xi^q\varpi_0)\right)\\&=\limsup_{p,q\to\infty}\left(\lambda+\Im(\mathcal{J}_d(\xi^p\varpi_0,\xi^q\varpi_0))\right)\\&\leq\limsup_{p,q\to\infty}\left(\Im(\delta_{p_0}(\mathcal{J}_d,\xi,\varpi_0))\right)\\&=\Im\left(\lim_{p_0\to\infty}\delta_{p_0}(\mathcal{J}_d,\xi,\varpi_0)\right)\\&=\Im(\mho),\end{aligned}\tag{3.5}$$

a contradiction because $\lambda>0$. Hence $\{\xi^p\varpi_0\}_{p\in\mathbb{N}}$ $\mathcal{J}_d$-Cauchy sequence. Since $(\mathcal{Z},\mathcal{J}_d)$ is $\sim_{\mathcal{R}}$-non-decreasing-complete, there is $\nu\in\mathcal{Z}$ such that $\{\varpi_p\}\xrightarrow{\mathcal{J}_d}\nu$. By using $(\mathcal{J}_{d3})$, we get

$$0\leq\mathcal{J}_d(\nu,\nu)\leq\kappa\limsup_{p\to\infty}\mathcal{J}_d(\varpi_p,\nu)=0,\tag{3.6}$$

which implies $\mathcal{J}_d(\nu,\nu)=0$.
Moreover, as we additionally assume that ξ is $\sim_{\mathcal{R}}$-non-decreasing-continuous, then

$$\{\varpi_{p+1}=\xi^p\varpi_0=\xi\varpi_p\}\xrightarrow{\mathcal{J}_d}\xi\nu.$$

Proposition 1.1 guarantees that $\xi\nu=\nu$. □

4. Consequences

In this section, we find more results involving stronger contractive conditions.

Corollary 4.1. *Let $(\mathcal{Z},\mathcal{J}_d)$ be a $\kappa_{\mathcal{JS}}$-MS with a preorder $\sim_{\mathcal{R}}$ and let $\xi:\mathcal{Z}\to\mathcal{Z}$ be an $\sim_{\mathcal{R}}$-non-decreasing mapping. Let $\varpi_0\in\mathcal{Z}$ be a point such that $\varpi_0\sim_{\mathcal{R}}\xi\varpi_0$ and $\delta p_0(\mathcal{J}_d,\xi,\varpi)<\infty$ for some $p_0\in\mathbb{N}$. Assume there is $b\in(0,1)$ such that*

$$\mathcal{J}_d(\xi\varpi,\xi\varsigma)\leq b\mathcal{Q}_\xi^{\mathcal{J}_d}(\varpi,\varsigma),\tag{4.1}$$

for all $\varpi,\varsigma\in\mathcal{O}_\xi(\varpi_0)$. Then the sequence $\{\xi^p\varpi_0\}_{p\in\mathbb{N}}$ based on ϖ_0 is $\sim_{\mathcal{R}}$-non-decreasing and $\mathcal{J}_d$-Cauchy sequence.
Furthermore, if $(\mathcal{Z},\mathcal{J}_d)$ is $\sim_{\mathcal{R}}$-non-decreasing-complete, then $\{\xi^p\varpi_0\}_{p\in\mathbb{N}}$ $\mathcal{J}_d$-converges to a point $\nu\in\mathcal{Z}$ that meets

$$\mathcal{J}_d(\nu,\nu)=0.$$

Additionally, if ξ is $\sim_{\mathcal{R}}$-non-decreasing-continuous, then $\nu\in\xi\nu$.

Proof. Define $\Im:(0,\infty)\to\mathbb{R}$ by $\Im(s)=\ln s$ for all $s\in(0,\infty)$. Put $b=\frac{1}{e^\lambda}$. Inequality (5.1) implies (2.5). Hence, all of the requirements of Theorem 3.1 have been met, and the proof is concluded. □

Corollary 4.2. *Let $(\mathcal{Z}, \mathcal{J}_d)$ be a $\kappa_{\mathcal{JS}}$-MS with a preorder $\sim_{\mathcal{R}}$ and let $\xi : \mathcal{Z} \to \mathcal{Z}$ be an $\sim_{\mathcal{R}}$-non-decreasing mapping. Let $\varpi_0 \in \mathcal{Z}$ be a point such that $\varpi_0 \sim_{\mathcal{R}} \xi\varpi_0$ and $\delta p_0(\mathcal{J}_d, \xi, \varpi) < \infty$ for some $p_0 \in \mathbb{N}$. Assume, there is a non-decreasing function $\Im : (0, \infty) \to (-\infty, \infty)$ fulfilling $(\Im_2')$, $(\Im_{\mathcal{J}_d})$*

$$\xi\varpi \neq \xi\varsigma \text{ implies } \lambda + \Im(\mathcal{J}_d(\xi\varpi, \xi\varsigma)) \leq \Im(\mathcal{Q}_\xi^{\mathcal{J}_d}(\varpi, \varsigma)), \text{ for all } \varpi, \varsigma \in \mathcal{Z} \text{ such that } \varpi \sim_{\mathcal{R}} \varsigma \quad (4.2)$$

for $\lambda > 0$. Then the sequence $\{\xi^p\varpi_0\}_{p\in\mathbb{N}}$ based on ϖ_0 is $\sim_{\mathcal{R}}$-non-decreasing and $\mathcal{J}_d$-Cauchy sequence.
Furthermore, if $(\mathcal{Z}, \mathcal{J}_d)$ is $\sim_{\mathcal{R}}$-non-decreasing-complete, then $\{\xi^p\varpi_0\}_{p\in\mathbb{N}}$ $\mathcal{J}_d$-converges to a point $\nu \in \mathcal{Z}$ that meets

$$\mathcal{J}_d(\nu, \nu) = 0.$$

Additionally, if ξ is $\sim_{\mathcal{R}}$-non-decreasing-continuous, then $\nu \in \xi\nu$.

Proof. Let the contractivity condition (5.2) hold for all $\varpi, \varsigma \in \mathcal{Z}$ such that $\varpi \sim_{\mathcal{R}} \varsigma$, then Lemma 2.1 guarantees that it also holds for $\varpi, \varsigma \in \mathcal{O}_\xi(\varpi_0)$. So due to Theorem 3.1, we obtained the result. □

Corollary 4.3. *Let $(\mathcal{Z}, \mathcal{J}_d)$ be a $\kappa_{\mathcal{JS}}$-MS with a preorder $\sim_{\mathcal{R}}$ and let $\xi : \mathcal{Z} \to \mathcal{Z}$ be an $\sim_{\mathcal{R}}$-non-decreasing mapping. Let $\varpi_0 \in \mathcal{Z}$ be a point such that $\varpi_0 \sim_{\mathcal{R}} \xi\varpi_0$ and $\delta p_0(\mathcal{J}_d, \xi, \varpi) < \infty$ for some $p_0 \in \mathbb{N}$. Assume, there is a non-decreasing continuous function $\Im : (0, \infty) \to (-\infty, \infty)$ fulfilling the inequality (5.2) for $\lambda > 0$ and the following:*

$$\lim_{p_0+\ell\to\infty} \delta_{p_0+\ell}(\mathcal{J}_d, \xi, \varpi_0) = \lim_{p_0\to\infty} \delta_{p_0}(\mathcal{J}_d, \xi, \varpi_0) \quad \text{for arbitrary } \ell \in \mathbb{N} \text{ such that } \ell \geq p_0.$$

Then the sequence $\{\xi^p\varpi_0\}_{p\in\mathbb{N}}$ based on ϖ_0 is $\sim_{\mathcal{R}}$-non-decreasing and $\mathcal{J}_d$-Cauchy sequence. Furthermore, if $(\mathcal{Z}, \mathcal{J}_d)$ is $\sim_{\mathcal{R}}$-non-decreasing-complete, then $\{\xi^p\varpi_0\}_{p\in\mathbb{N}}$ $\mathcal{J}_d$-converges to a point $\nu \in \mathcal{Z}$ that meets

$$\mathcal{J}_d(\nu, \nu) = 0.$$

Additionally, if ξ is $\sim_{\mathcal{R}}$-non-decreasing-continuous, then $\nu \in \xi\nu$.

Proof. By using the same reason as in the proof of Corollary 4.2, Theorem 3.2 gives the result. □

Corollary 4.4. *Let $(\mathcal{Z}, \mathcal{J}_d)$ be a $\kappa_{\mathcal{JS}}$-MS with a partial order $\ll$ and let $\xi : \mathcal{Z} \to \mathcal{Z}$ be an $\ll$-non-decreasing mapping. Let $\varpi_0 \in \mathcal{Z}$ be a point such that $\varpi_0 \ll \xi\varpi_0$ and $\delta p_0(\mathcal{J}_d, \xi, \varpi) < \infty$ for some $p_0 \in \mathbb{N}$. Assume, there is a non-decreasing function $\Im : (0, \infty) \to (-\infty, \infty)$ satisfying $(\Im_2')$, $(\Im_{\mathcal{J}_d})$ and (2.5) for all $\varpi, \varsigma \in \mathcal{O}_\xi(\varpi_0)$ and $\lambda > 0$. Then, the sequence $\{\xi^p\varpi_0\}_{p\in\mathbb{N}}$ based on ϖ_0 is $\ll$-non-decreasing and $\mathcal{J}_d$-Cauchy sequence.*
Furthermore, if $(\mathcal{Z}, \mathcal{J}_d)$ is $\ll$-non-decreasing-complete, then $\{\xi^p\varpi_0\}_{p\in\mathbb{N}}$ $\mathcal{J}_d$-converges to a point $\nu \in \mathcal{Z}$ that meets

$$\mathcal{J}_d(\nu, \nu) = 0.$$

Additionally, if ξ is $\ll$-non-decreasing-continuous, then $\nu \in \xi\nu$.

Proof. Due to the fact that a partial order $\ll$ is a preorder $\sim_{\mathcal{R}}$, the conclusion is reached by applying Theorem ref 3.1. $\square$

Corollary 4.5. *Let $(\mathcal{Z}, \mathcal{J}_d)$ be a $\kappa_{\mathcal{JS}}$-MS with a partial order $\ll$ and let $\xi : \mathcal{Z} \to \mathcal{Z}$ be an $\ll$-non-decreasing mapping. Let $\varpi_0 \in \mathcal{Z}$ be a point such that $\varpi_0 \ll \xi\varpi_0$ and $\delta p_0(\mathcal{J}_d, \xi, \varpi) < \infty$ for some $p_0 \in \mathbb{N}$. Assume, there is a non-decreasing continuous function $\Im : (0, \infty) \to (-\infty, \infty)$ satisfying (2.5) for all $\varpi, \varsigma \in \mathcal{O}_\xi(\varpi_0)$, $\lambda > 0$ and the following:*

$$\lim_{p_0+\ell\to\infty} \delta_{p_0+\ell}(\mathcal{J}_d, \xi, \varpi_0) = \lim_{p_0\to\infty} \delta_{p_0}(\mathcal{J}_d, \xi, \varpi_0) \quad \textit{for arbitrary } \ell \in \mathbb{N} \textit{ such that } \ell \geq p_0.$$

Then the sequence $\{\xi^p\varpi_0\}_{p\in\mathbb{N}}$ based on ϖ_0 is $\ll$-non-decreasing and $\mathcal{J}_d$-Cauchy sequence. Furthermore, if $(\mathcal{Z}, \mathcal{J}_d)$ is $\ll$-non-decreasing-complete, then $\{\xi^p\varpi_0\}_{p\in\mathbb{N}}$ $\mathcal{J}_d$-converges to a point $\nu \in \mathcal{Z}$ that meets

$$\mathcal{J}_d(\nu, \nu) = 0.$$

Additionally, if ξ is $\ll$-non-decreasing-continuous, then $\nu \in \xi\nu$.

Proof. Due to the fact that a partial order $\ll$ is a preorder $\sim_{\mathcal{R}}$, the conclusion is reached by applying Theorem ref 3.2. $\square$

Corollary 4.6. *Let $(\mathcal{Z}, \mathcal{J}_d)$ be a $\kappa_{\mathcal{JS}}$-MS with a preorder $\sim_{\mathcal{R}}$ and let $\xi : \mathcal{Z} \to \mathcal{Z}$ be an $\sim_{\mathcal{R}}$-non-decreasing mapping. Let $\varpi_0 \in \mathcal{Z}$ be a point such that $\varpi_0 \sim_{\mathcal{R}} \xi\varpi_0$ and $\delta p_0(\mathcal{J}_d, \xi, \varpi) < \infty$ for some $p_0 \in \mathbb{N}$. Assume, there is a non-decreasing function $\Im : (0, \infty) \to (-\infty, \infty)$ satisfying $(\Im_2')$, $(\Im_{\mathcal{J}_d})$ and*

$$\lambda + \Im(\mathcal{J}_d(\xi\varpi, \xi\varsigma)) \leq \Im\left(\max\{\mathcal{J}_d(\varpi, \varsigma), \frac{\mathcal{J}_d(\varpi, \xi\varpi) + \mathcal{J}_d(\varsigma, \xi\varsigma)}{2}, \frac{\mathcal{J}_d(\varpi, \xi\varsigma) + \mathcal{J}_d(\varsigma, \xi\varpi)}{2}\}\right) \tag{4.3}$$

for all $\varpi, \varsigma \in \mathcal{O}_\xi(\varpi_0)$ with $\xi\varpi \neq \xi\varsigma$ and $\lambda > 0$. Then the sequence $\{\xi^p\varpi_0\}_{p\in\mathbb{N}}$ based on ϖ_0 is $\sim_{\mathcal{R}}$-non-decreasing and $\mathcal{J}_d$-Cauchy sequence.
Furthermore, if $(\mathcal{Z}, \mathcal{J}_d)$ is $\sim_{\mathcal{R}}$-non-decreasing-complete, then $\{\xi^p\varpi_0\}_{p\in\mathbb{N}}$ $\mathcal{J}_d$-converges to a point $\nu \in \mathcal{Z}$ that satisfies

$$\mathcal{J}_d(\nu, \nu) = 0.$$

Additionally, if ξ is $\sim_{\mathcal{R}}$-non-decreasing-continuous, then $\nu \in \xi\nu$.

Proof. Since $\frac{(r+s)}{2} \leq \max\{s, r\}$ for all $s, r \in [0, \infty]$, so inequality (5.3) implies inequality (2.5) and Theorem 3.1 leads to the conclusion. $\square$

Corollary 4.7. *Let $(\mathcal{Z}, \mathcal{J}_d)$ be a $\kappa_{\mathcal{JS}}$-MS with a preorder $\sim_{\mathcal{R}}$ and let $\xi : \mathcal{Z} \to \mathcal{Z}$ be an $\sim_{\mathcal{R}}$-non-decreasing mapping. Let $\varpi_0 \in \mathcal{Z}$ be a point such that $\varpi_0 \sim_{\mathcal{R}} \xi\varpi_0$ and $\delta p_0(\mathcal{J}_d, \xi, \varpi) < \infty$ for some $p_0 \in \mathbb{N}$. Assume, there is a continuous non-decreasing function $\Im : (0, \infty) \to (-\infty, \infty)$ obeying*

$$\lambda + \Im(\mathcal{J}_d(\xi\varpi, \xi\varsigma)) \leq \Im\left(\max\{\mathcal{J}_d(\varpi, \varsigma), \mathcal{J}_d(\varpi, \xi\varpi), \mathcal{J}_d(\varsigma, \xi\varsigma), \frac{\mathcal{J}_d(\varpi, \xi\varsigma) + \mathcal{J}_d(\varsigma, \xi\varpi)}{2}\}\right), \tag{4.4}$$

for all $\varpi, \varsigma \in \mathcal{O}_\xi(\varpi_0)$ with $\xi\varpi \neq \xi\varsigma$, $\lambda > 0$ and the following:

$$\lim_{p_0+\ell\to\infty} \delta_{p_0+\ell}(\mathcal{J}_d, \xi, \varpi_0) = \lim_{p_0\to\infty} \delta_{p_0}(\mathcal{J}_d, \xi, \varpi_0) \quad \textit{for arbitrary } \ell \in \mathbb{N} \textit{ such that } \ell \geq p_0.$$

Then the sequence $\{\xi^p \varpi_0\}_{p\in\mathbb{N}}$ *based on* ϖ_0 *is* $\sim_{\mathcal{R}}$*-non-decreasing and* $\mathcal{J}_d$*-Cauchy sequence. Furthermore, if* $(\mathcal{Z}, \mathcal{J}_d)$ *is* $\sim_{\mathcal{R}}$*-non-decreasing-complete, then* $\{\xi^p \varpi_0\}_{p\in\mathbb{N}}$ $\mathcal{J}_d$*-converges to a point* $\nu \in \mathcal{Z}$ *that obeys*

$$\mathcal{J}_d(\nu, \nu) = 0.$$

Additionally, if ξ *is* $\sim_{\mathcal{R}}$*-non-decreasing-continuous, then* $\nu \in \xi\nu$.

Proof. Since $\frac{(r+s)}{2} \leq \max\{s, r\}$ for all $s, r \in [0, \infty]$, so inequality (5.3) implies inequality (2.5) and the result follows from Theorem 3.2. □

Remark 4.1. Theorem 3.1, Theorem 3.2 and Corollaries 4.1-4.7 remain true if we do one or more of the following changes in their statement:

- exchange the $\sim_{\mathcal{R}}$-non-decreasing-completeness of $(\mathcal{Z}, \mathcal{J}_d)$ by the completeness of $(\mathcal{Z}, \mathcal{J}_d)$;
- exchange the $\sim_{\mathcal{R}}$-non-decreasing-continuity of ξ by continuity;
- exchange the preorder $\sim_{\mathcal{R}}$ by the trivial preorder $\sim_{\mathcal{R}_{\mathcal{Z}}}$ given by $\varpi \sim_{\mathcal{R}_{\mathcal{Z}}} \varsigma$ for all $\varpi, \varsigma \in \mathcal{Z}$;
- exchange, in the contractivity condition, for all $\varpi, \varsigma \in \mathcal{O}_\xi(\varpi_0)$ by for all $\varpi, \varsigma \in \mathcal{Z}$ such that $\varpi \sim_{\mathcal{R}} \varsigma$;
- exchange the $\kappa_{\mathcal{JS}}$-MS by any of the abstract metric spaces that could be considered as a $\kappa_{\mathcal{JS}}$-MS: b-metric spaces, modular spaces, and standard metric spaces.
- exchange the contractivity condition (2.5) by

  $$\lambda + \Im(\mathcal{J}_d(\xi\varpi, \xi\varsigma)) \leq \Im\left(\mathcal{J}_d(\varpi, \varsigma)\right),$$

  for every $\varpi, \varsigma \in \mathcal{O}_\xi(\varpi_0)$ with $\xi\varpi \neq \xi\varsigma$.

5. Existence of solution to RLC circuit's current differential equation

A tuning circuit is a mathematical representation of the electric current in an RLC parallel circuit to presented with a rudimentary knowledge of how light is converted into electricity. Consider the following series of electric circuit (Figure ??), which includes a resistor R, a capacitor C, an inductor L, a voltage V, and an electromotive force E. With the aid of Kirchhoff's Voltage Law, related problems are mathematically modelled as initial value problems for second order ordinary differential equations of the form:

$$\begin{cases} \mathrm{L}\frac{d^2 q}{de^2} + \mathrm{R}\frac{dq}{de} + \frac{q}{\mathrm{C}} = \mathrm{V}_v(e) \\ \\ q(0) = q'(0) = 0, \end{cases} \tag{5.1}$$

where $\mathrm{V}_v(e) = \mathrm{V}$.

In this part, we demonstrate the existence of the solution to the RLC differential equation (5.1). The problem (5.1) is identical to the following integral equation:

$$q(e) = \int_0^e \mathcal{G}(e, g)\hbar(g, q(g))dg, \quad e \in [0, 1], \tag{5.2}$$

where $\hbar : [0,1] \times \mathbb{R} \to \mathbb{R}$ is a monotonically non-decreasing function for all $g \in [0,1]$ and $\mathcal{G}$ is the Green function defined as

$$\mathcal{G}(e,g) = \begin{cases} -ge^{-\tau(g-e)} & \text{if } 0 \le g \le e \le 1; \\ -ee^{-\tau(g-e)} & \text{if } 0 \le e \le g \le 1, \end{cases} \tag{5.3}$$

τ is a constant computed in terms of R and L. Let $\mathcal{Z} = C(J,\mathbb{R})$ be the space of all continuous real valued functions on J, where $J = [0,1]$. Then $\mathcal{Z}$ is a complete metric space with respect to the metric $\mathcal{J}_d(\hat{a},\hat{b}) = \|\hat{a} - \hat{b}\|_\infty = \sup_{e\in J} |\hat{a}(e) - \hat{b}(e)|$ and so $\mathcal{Z}$ is $\kappa_{\mathcal{JS}}$-MS for $\kappa = 1$. Hereafter, we assume that $(\mathcal{Z},\mathcal{J}_d)$ is a $\kappa_{\mathcal{JS}}$-MS with canonical preorder $\preceq$ and $(\mathcal{Z},\mathcal{J}_d)$ is $\preceq$-non-decreasing-complete. Define the operator $\aleph : \mathcal{Z} \to \mathcal{Z}$ as follows:

$$\aleph(q(e)) = \int_0^e \mathcal{G}(e,g)\hbar(g,q(g))dg, \quad e \in [0,1]. \tag{5.4}$$

A fixed point of operator (5.4) is the solution of problem (5.1). We take into account the following hypotheses:

(H1) $\mathcal{G} : J^2 \to [0,\infty)$ is a continuous function;
(H2) $|\hbar(e,q(e)) - \hbar(e,p(e))| \le |q(e) - p(e)| + 1$ for all $e \in [0,1]$;
(H3) $\|\mathcal{G}\|_\infty = \sup_{e\in I} \int_0^e \mathcal{G}(e,g) \le 1$;
(H4) $\aleph$ is $\preceq$-non-decreasing continuous.

THEOREM 5.1. *Suppose that hypothesis* $(H1)$-$(H4)$ *hold. Then, the initial value problem* (5.1) *has a common solution in* $\mathcal{Z}$*.*

Proof. Firstly, note that for $\Im(\hat{a}) = \frac{1}{\hat{a}+1}$ for all $\hat{a} \in (0,\infty)$, inequality (2.5) is equivalent to the following for all $\varpi,\varsigma \in \mathcal{Z}$:

$$\mathcal{J}_d(\xi\varpi,\xi\varsigma) \le \frac{\mathcal{Q}_\xi^{\mathcal{J}_d}(\varpi,\varsigma)+1}{1+\lambda\mathcal{Q}_\xi^{\mathcal{J}_d}(\varpi,\varsigma)} - 1 \le \frac{\mathcal{Q}_\xi^{\mathcal{J}_d}(\varpi,\varsigma)+1}{1+\lambda\mathcal{Q}_\xi^{\mathcal{J}_d}(\varpi,\varsigma)} \le \mathcal{Q}_\xi^{\mathcal{J}_d}(\varpi,\varsigma)+1. \tag{5.5}$$

Next, for all $p,q \in \varnothing_\aleph(p_0)\mathcal{Z}$ and $e \in J$, we have

$$\begin{aligned} |\aleph(q(e)) - \aleph(p(e))| &= \left| \int_0^e \mathcal{G}(e,g)[\hbar(g,q(g)) - \hbar(g,p(g))]dg \right| \\ &\le \int_0^e \mathcal{G}(e,g)\,|[\hbar(g,q(g)) - \hbar(g,p(g))]|\,dg \\ &\le \int_0^e \mathcal{G}(e,g)|q(e) - p(e)|dg \\ &\le \int_0^e \mathcal{G}(e,g)(\max\{|q(e)-p(e)|, |\aleph(p(e))-p(e)|, |\aleph(q(e))-q(e)|, \\ &\quad |\aleph(q(e))-p(e)|, |\aleph(p(e))-q(e)|\}+1)dg, \end{aligned}$$

This implies that

$$\begin{aligned} \|\aleph(q)-\aleph(p)\|_\infty \le& \|\mathcal{G}\|_\infty \left(\max\{\|p-q\|_\infty, \|\aleph(p)-p\|_\infty, \|\aleph(q)-q\|_\infty, \|\aleph(q)-p\|_\infty,\right. \\ & \left.\|\aleph(p)-q\|_\infty\}+1\right). \end{aligned} \tag{5.6}$$

From (H3) and (5.6), we have

$$\|\aleph(q)-\aleph(p)\|_\infty \leq \max\{\|p-q\|_\infty, \|\aleph(p)-p\|_\infty, \|\aleph(q)-q\|_\infty, \|\aleph(q)-p\|_\infty, \|\aleph(p)-q\|_\infty\}+1.$$

Hence (2.5) is satisfied for $\Im(\hat{a}) = -\frac{1}{\hat{a}+1}$. Thus, all hypotheses of Theorem 3.2 are satisfied and therefore differential equation (5.1) has a solution in J. □

Conclusion

In this work, we establish weak $\Im$-contractions and show some fixed point theorems for these contractive conditions in the JS generalized metric spaces. Finally, we proved fixed point results, an existence result for the solution of the RLC circuit's current differential equation is also established.

Acknowledgment

Authors are thankful to the editor and anonymous referees for their valuable comments and suggestions.

Funding

This research received no external funding.

Data Availability

The data used to support the findings of this study are available from the corresponding author upon request.

Conflicts of Interest

The authors declare no conflict of interest.

Author Contributions

Conceptualization, B.I., N.S. and S.R., Formal analysis, N.S., B.I. Investigation, N.S., B.I., S.R. Writing original draft preparation, B.I. and N.S. Writingreview and editing, N.S., and R.G. All authors have read and agreed to the published version of the manuscript.

References

[1] M. Abbas, T. Nazir, T. A. Lampert, S. Radenović, *Common fixed points of set-valued F-contraction mappings on domain of sets endowed with directed graph*, Comput. Appl. Math., (2016), 1–16.
[2] I. Altun, N. Al Arif, M. Jleli, A. Lashin, B. Samet, *Feng-Liu type fixed point results for multivalued mappings on JS-metric spaces*, J. Nonlinear Sci. Appl., **9** (2016), 3892–3897.
[3] S. Banach, *Sur les opérations dans les ensembles abstraits et leur application aux équations intégrales*, Fund. Math., **3** (1922), 133–181.
[4] S. Czerwik, *Contraction mappings in b-metric spaces*, Acta Math. Inform. Univ. Ostrav., **1** (1993), 5–11.
[5] D. Dumitrescu, A. Pitea, *Fixed point theorems on almost (ϕ,θ)-contractions in Jleli-Samet generalized metric spaces*, Mathematics, **10(22)** (2022), 4239.

[6] D. Derouiche, H. Ramoul, *New fixed point results for F-contractions of Hardy-Rogers type in b-metric spaces with applications*, J. Fixed Point Theory Appl., **22** (2020), 86.
[7] D. Gopal, M. Abbas, D. K. Patel, C. Vetro, *Fixed points of α-type F-contractive mappings with an application to nonlinear fractional differential equation*, Acta Math. Sci., **36** (2016), 957–970.
[8] N. Hussain, A. Latif, I. Iqbal, *Fixed point results for generalized F-contractions in modular metric and fuzzy metric spaces*, Fixed Point Theory and Appl., **2015** (2015), 158.
[9] N. Hussain, P. Salimi, *Suzuki-Wardowski type fixed point theorems for α-GF-contractions*, Taiwan. J. Math., **18** (2014), 1879–1895.
[10] I. Iqbal, N. Hussain, *Fixed point theorems for generalized multivalued nonlinear F-contractions*, J. Nonlinear Sci. Appl., **9** (2016), 5870–5893.
[11] I. Iqbal, N. Hussain, N. Sultana, *Fixed Points of multivalued non-linear F-contractions with application to solution of matrix equations*, Filomat, **31(11)** (2017), 3319–3333.
[12] B. Iqbal, N. Saleem, I. Iqbal, R. George, *Common and Coincidence Fixed Point Theorems for $\Im$-Contractions with Existence Results for Nonlinear Fractional Differential Equations*, Fractal Fract., **2023** (2023), 47.
[13] P. Hitzler, A. K. Seda, *Dislocated topologies*, J. Electr. Eng., **51(12)** (2000), 3–7.
[14] M. Jleli, B. Samet, *A generalized metric space and related fixed point theorems*, Fixed Point Theory Appl., **2015** (2015), 61 pages.
[15] E. Karapinar, D. O'Regan, A. F. Rold*á*n López de Hierro, N. Shahzad, *Fixed point theorems in new generalized metric spaces*, J. Fixed Point Theory Appl., **18** (2016), 645–671.
[16] A. A. Kilbas, H. M. Sirivastava, J. J. Turjillo, *Theory and applications of fractional differential equations*, North-Holland Mathematics Studies, Elsevier, Amsterdam, The Netherlands, (2006).
[17] A. Lukács, S. Kajáantó, *On the conditions of fixed-point theorems concerning F-contractions*, Results Math., **73** (2018), 82.
[18] J. Musielak, W. Orlicz, *On modular spaces*, Stud. Math, **18** (1959), 49–65.
[19] J. Musielak, W. Orlicz, *Some remarks on modular spaces*, Bull. Acad. Polon. Sci. Sr. Sci. Math. Astron. Phys., **7** (1959), 661–668.
[20] Z. D. Mitrović, S. Radenović, H. Aydi, A. A. Altasan, C. Ozel, *On two new approaches in modular spaces*, Ital. J. Pure Appl. Math., **41** (2019), 679–690.
[21] H. Nakano, *Modular semi-ordered spaces*, Tokyo, (1950).
[22] P. D. Proinov, *Fixed point theorems for generalized contractive mappings in metric spaces*, J. Fixed Point Theory Appl., **22** (2020), 21.
[23] H. Piri, P. Kumam, *Some fixed point theorems concerning F-contraction in complete metric spaces*, Fixed Point Theory Appl., **2014** (2014), 210.
[24] A. C. M. Ran, M. C. B. Reurings, *A fixed point theorem in partially ordered sets and some applications to matrix equations*, Proc. Am. Math. Soc., **132** (2004), 1435–1443.
[25] N. Saleem, I. Iqbal, B. Iqbal, S.Radenović, *Coincidence and fixed points of multivalued F-contractions in generalized metric space with application*, J. Fixed Point Theory Appl., **22** (2020), 81.
[26] N. A. Secelean, *Iterated function systems consisting of F-contractions*, Fixed Point Theory Appl., **2013** (2013), 277.
[27] N. Shahzad, E. Karapnar, A. F. Roldán-López-de-Hierro, *On some fixed point theorems under (α, ψ, ϕ)-contractivity conditions in metric spaces endowed with transitive binary relations*, Fixed Point Theory Appl., **2015** (2015), 124.
[28] V. Todorčević, *Harmonic quasiconformal mappings and hyperbolic type metric*, Springer Nature, Switzerland AG, (2019).
[29] F. Vetro, *F-contractions of Hardy-Rogers type and application to multistage decision processes*, Nonlinear Anal. Model. Control, **21** (2016), 531–546.
[30] D. Wardowski, *Fixed points of a new type of contractive mappings in complete metric spaces*, Fixed Point Theory and Appl., **2012** (2012), 94.
[31] D. Wardowski, *Solving existence problems via F-contractions*, Proc. Am. Math. Soc., **146** (2018), 1585–1598.
[32] D. Wardowski, N. Van Dung, *Fixed points of F-weak contractions on complete metric space*, Demonstratio Math., **2014** (2014), 146–155.

 Click here to access/download;Figure;Fig.png

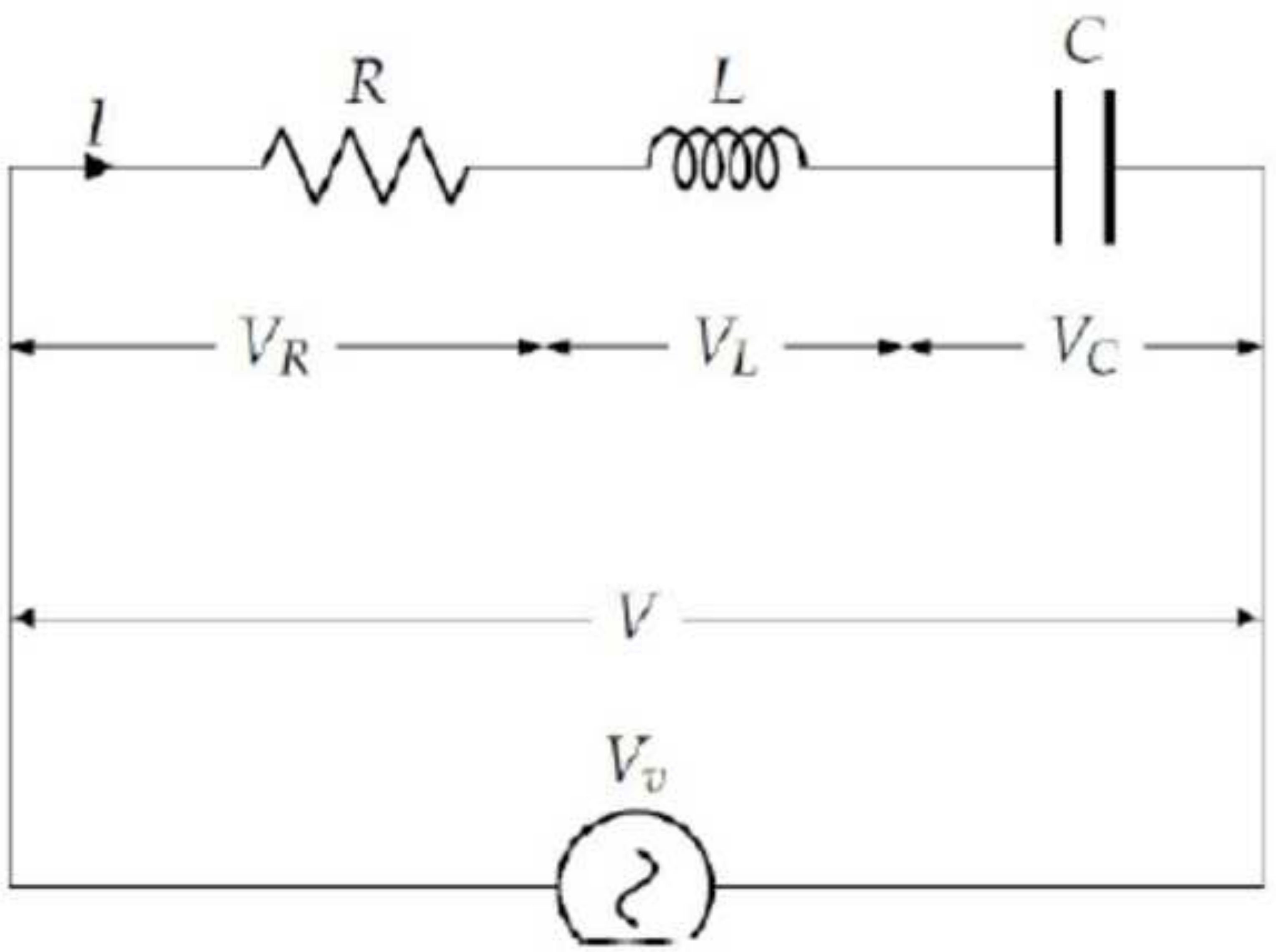

FIGURE1.RLC-SeriesCircuit
