# Supplementary material for: Fixed point results for ℑ-Contractions in JS-generalized metric spaces with an application
Source: PLoS One. 2025 Feb 18;20(2):e0314493. doi: 10.1371/journal.pone.0314493 (PMC11835342; doi:10.1371/journal.pone.0314493)
Supplement: Attachment — Submitted filename: Report-Saleem-F-contraction.pdf [file pone.0314493.s003.pdf]

A Report of the manuscript entitled

Fixed Point Results for Weak $\Im$-Contractions in JS Generalized Metric Spaces with an Application

In this manuscript, authors present an existence result for the solution of the RLC circuit's current differential equation and for this they firstly prove some fixed point theorems for different weak contractive conditions in the domain of JS generalized metric spaces. The findings given in this paper seem correct and interesting but following points are notable:

- The main difference between Theorem 3.1 and Theorem 3.2 is the choice of the condition on function $\Im$. So add at least one example in the manuscript which shows that there exist functions which satisfy one condition but not another.
- At page 2, in introduction authors used the terminology of JS generalized metric space define by Jleli and Samet but did not defined this earlier.
- There are some typing and formatting mistakes in whole manuscript so revise it carefully.

In the light of above remarks, I recommend Major Revision of the manuscript.
