# Supplementary material for: Fixed point results for ℑ-Contractions in JS-generalized metric spaces with an application
Source: PLoS One. 2025 Feb 18;20(2):e0314493. doi: 10.1371/journal.pone.0314493 (PMC11835342; doi:10.1371/journal.pone.0314493)
Supplement: Attachment — Submitted filename: Response to reviewers.pdf [file pone.0314493.s004.pdf]

Reply to Reviewer 1

(1) The main difference between Theorem 3.1 and Theorem 3.2 is the choice of the condition on function $\Im$. So add at least one example in the manuscript which shows that there exist functions which satisfy one condition but not another.

Answer: Example 3.1 is added in the revised manuscript.

(2) At page 2, in introduction authors used the terminology of JS generalized metric space define by Jleli and Samet but did not defined this earlier.

Answer: In revised manuscript, JS-generalized metric space is now firstly defined at page 2.

(3) There are some typing and formatting mistakes in whole manuscript so revise it carefully.

Answer: The paper is carefully revised and corrected as per suggestions and instructions given by the reviewers.

Reply to Reviewer 2

(1) On page 2, replace in numerous ways. (see [4, 13, 18, 19, 20, 21, 24, 28]) with in numerous ways (see [4, 13, 18, 19, 20, 21, 24, 28]).

Answer: Corrected in the revised version.

(2) Add a potential example of the theoretical results (Main Result) for the understanding of the readers and to show the applicability.

Answer: Example 3.2 is added in the revised manuscript.

(3) At page 2, in 3rd paragraph, add space in the following: in[27]., Hereafter.

Answer: Corrected.

(4) In Remark 1.3, replace complete As with complete. As

Answer: Replaced as suggested.

(5) There are several other typo mistakes in the article, authors are encouraged to revise the article as per suggestions.

Answer: The paper is carefully revised and corrected as per suggestions and instructions given by the reviewers. All mistakes are addressed and corrected accordingly.

Reply to Reviewer 3

All comments mentioned in the Referee's report are addressed as follows:

(1) Yes, this is of weak type. In honor of referee's, we have omitted the word weak from the Title.

(2) JS generalized metric space is replaced by JS-generalized metric space in whole revised manuscript.

(3) In Abstract, domain is replaced by setting.

(4) RLC is an acronym that stands for the three components of the circuit. R is for the resistor. L is for the inductor. C is for the capacitor and this is described in Section 5 of the manuscript.

(5) The interval in Lemma 1.1 is corrected.

(6) References are cited in suggested place.

(7) Notations and symbols are improved and changed to avoid any ambiguity and confusion. The revised paper is now more readable and transparent.

(8) The paper is carefully revised and corrected as per suggestions and instructions mentioned by the reviewer. All mistakes are addressed and corrected accordingly.

(9) In the light of remark 1.3, Example 1.2 is actually a counter example, which shows that a $\sim_R$-non-decreasing complete metric space need not to be complete.

(10) The integral equation (5.2) is obtained from initial value problem (5.1) by taking integral twice and using initial conditions and already obtained in [20] and [21]. These papers are cited in the revised manuscript.

(11) To prove Theorem 5.1, we used Theorem 3.2, in which $\Im : (0, \infty) \to \mathbb{R}$ is a non-decreasing and continuous function. The function $\Im : (0, \infty) \to \mathbb{R}$ defined by $\Im(a) = -\frac{1}{a+1}$ is non-decreasing and continuous, so we can consider it in Theorem 5.1.
